# Firing-rate models for neurons with a broad repertoire of spiking behaviors

**DOI:** 10.1007/s10827-018-0693-9

**Published:** 2018-08-27

**Authors:** Thomas Heiberg, Birgit Kriener, Tom Tetzlaff, Gaute T. Einevoll, Hans E. Plesser

**Affiliations:** 1 Faculty of Science and Technology, Norwegian University of Life Sciences, Ås, Norway; 2 Institute of Neuroscience and Medicine (INM-6), Jülich Research Centre, Jülich, Germany; 3 Institute for Advanced Simulation (IAS-6), Jülich Research Centre, Jülich, Germany; 4 JARA Institute Brain Structure-Function Relationships (INM-10), Jülich Research Centre, Jülich, Germany; 5 Department of Physics, University of Oslo, Oslo, Norway; 6 Present Address: Institute of Basic Medical Sciences, University of Oslo, Oslo, Norway

**Keywords:** Rate model, Linear-nonlinear model, Izhikevich model, AMAT model

## Abstract

Capturing the response behavior of spiking neuron models with rate-based models facilitates the investigation of neuronal networks using powerful methods for rate-based network dynamics. To this end, we investigate the responses of two widely used neuron model types, the Izhikevich and augmented multi-adapative threshold (AMAT) models, to a range of spiking inputs ranging from step responses to natural spike data. We find (i) that linear-nonlinear firing rate models fitted to test data can be used to describe the firing-rate responses of AMAT and Izhikevich spiking neuron models in many cases; (ii) that firing-rate responses are generally too complex to be captured by first-order low-pass filters but require bandpass filters instead; (iii) that linear-nonlinear models capture the response of AMAT models better than of Izhikevich models; (iv) that the wide range of response types evoked by current-injection experiments collapses to few response types when neurons are driven by stationary or sinusoidally modulated Poisson input; and (v) that AMAT and Izhikevich models show different responses to spike input despite identical responses to current injections. Together, these findings suggest that rate-based models of network dynamics may capture a wider range of neuronal response properties by incorporating second-order bandpass filters fitted to responses of spiking model neurons. These models may contribute to bringing rate-based network modeling closer to the reality of biological neuronal networks.

## Introduction

The simulation of large networks of spiking neurons on the scale of cortical columns or even whole areas of the cortex has become feasible due to advances in computer technology and simulator software (Helias et al. 2012; Kunkel et al. 2014). In order to relate simulation results to experimental findings, it is important to employ neuron models that accurately capture actual neuron dynamics in response to realistic stimuli. Dynamical models that reproduce the responses of individual neurons to injected currents go back to the seminal work by Hodgkin and Huxley (1952). Their conductance-based model quantitatively described the action potential initiation and propagation in the squid giant axon in response to depolarizing currents and spawned many variants and simplifications that have been analyzed and used in computational neuroscience ever since. Examples are the FitzHugh (1961) and the Morris-Lecar model (Morris and Lecar 1981). On the more abstract side of neuron modeling, Lapicque’s neuron model (Lapicque 1907), widely known as the leaky integrate-and-fire (IAF) neuron, models the membrane potential *V* (*t*) as a passive current integrator with leak current, emitting a spike whenever *V* (*t*) reaches a threshold value *𝜃*, followed by a membrane potential reset (Tuckwell 1988; Burkitt 2006a, 2006b).

These simple integrate-and-fire neuron models have particular appeal to computational neuroscientists because they capture the essential function of a neuron, while still being amenable to mathematical analysis in many input and network scenarios.

**Fig. 1:**
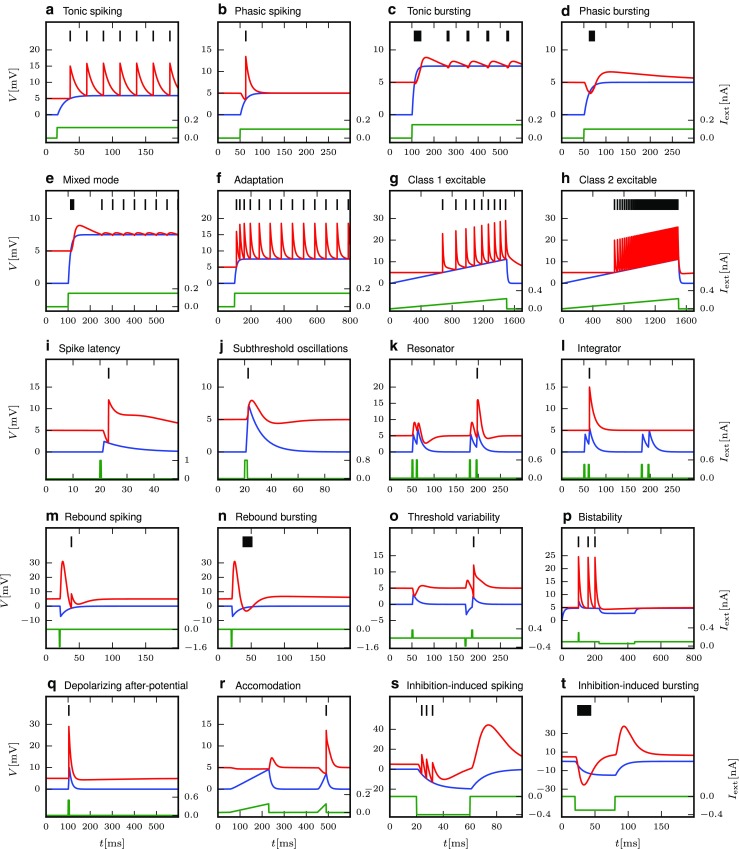
Response types for current input as defined by Izhikevich (2004). This illustration was created with our NEST implementation of the augmented MAT model (Yamauchi et al. 2011). Some model and stimulus parameters differ from those given by Yamauchi et al. (2011), see the Appendix. Membrane potential *V*_m_ is shown in blue, the threshold *V*_th_ in red, and the input current in green, while emitted spikes are shown as black bars; after Yamauchi et al. (2011). Subfigures are labeled as in Izhikevich (2004, Fig. 1)

Yet, the ideal model would be a neuron model that is both simple in its dynamical equations and still captures most of the actual response dynamics of a real neuron to a wide range of stimuli. To this end, Izhikevich suggested a two-dimensional neuron model that is able to reproduce at least twenty different characteristic responses that are commonly used to classify neuron response types in experiments, such as tonic, phasic and rebound spiking and bursting, or adaptation (Izhikevich 2010). The response types are illustrated in Fig. 1. The stimuli used to induce these spiking behaviors are direct current injections, ramp current injections or short direct current steps or pulses as indicated at the bottom of all panels.

In a network context, however, neurons usually receive noisy input currents. Moreover, they are known to respond highly reliably to repeated injections of the same frozen noise injection, while responses vary widely across trials when neurons receive identical direct current (Mainen and Sejnowski 1996). Neurons thus respond stereotypically to certain temporal input features rather than to mere current amplitude.

Motivated by such findings, Gerstner and colleagues showed that nonlinear IAF models, including the spike-response model and the adaptive exponential IAF model, can succesfully be mapped to experimental spike data in a noisy input regime and even have good spike-time prediction power (Brette and Gerstner 2005; Jolivet et al. 2006). Yet, the nonlinearity and the number of parameters in general make fitting a difficult task. The *International Competition on Quantitative Neuron Modeling* has challenged modelers to fit their neuron models to a set of spike data recorded from neurons stimulated with noisy input currents (Jolivet et al. 2008). The resulting neuron models were tested with a noisy input current that was not included in the training set, and the predicted spike times were compared to those of the actually emitted spikes. The *multi-timescale adaptive threshold model* (MAT model) introduced by Kobayashi et al. (2009), a surprisingly simple model with linear subthreshold dynamics, solved this task best. Despite its simplicity, the MAT model can generate type-I and type-II excitability, as well as burst firing. Moreover, an extended version of the MAT model, the *augmented MAT* (AMAT) model, which incorporates threshold dynamics that depend on the membrane-potential history, is able to reproduce all twenty spike response patterns described for the Izhikevich model (Yamauchi et al. 2011). Because of its few parameters and simple dynamics, the AMAT model has low computational cost while providing a large dynamical repertoire, and is thus highly attractive for large-scale network simulations.

In an actual neuronal network, neurons typically integrate spikes from thousands of presynaptic neurons, yet not all spikes might necessarily have a strong impact on the membrane potential. In many spiking network models, the effect of individual spikes on the membrane potential is assumed to be small, and spiking activity asynchronous and irregular. In this limit it is indeed possible to substitute the input current by, e.g., Gaussian white noise or an Ornstein-Uhlenbeck process (Johannesma 1968). However, experimental findings have repeatedly demonstrated that, even though most synapses are weak, synaptic weight distributions typically have heavy tails, with some corresponding to post-synaptic potentials of up to 10 mV (Song et al. 2005; Lefort et al. 2009; Avermann et al. 2012; Ikegaya et al. 2013). It is thus important to extend the analysis of neuronal response dynamics to input spike trains that elicit large individual post-synaptic potentials.

At an even higher level of abstraction are models that ignore specific spike times and heterogeneities in network structure, i.e., rate and field models. In contrast to high-dimensional networks of spiking neurons, such models are often easier to analyze mathematically due to their low dimensionality, and hence can offer insight into steady states of network activity and bifurcations that give rise to complex spatio-temporal phenomena, such as oscillatory dynamics, traveling waves or activity bump formation. Prominent examples are neural mass models, such as the Jansen-Rit model (Jansen and Rit 1995), and neural field models, such as the Wilson-Cowan model (Wilson and Cowan 1972), which include spatial interactions between neurons. In these models, the dynamics of large, possibly heterogeneous, populations of neurons are substituted by rate variables in a mean-field manner (Ermentrout 1998; Coombes 2005).

An important conceptual step in the derivation of these models is the substitution of the spiking activity of a neuron in response to a certain input current *I*(*t*) by an appropriate rate function^1^ mapping the input history {*I*(*s*)|*s* ≤ *t*} to the response rate at time *t*. Common choices are abstract models such as threshold-linear or sigmoidal functions *F*(*I*(*t*)) depending only on the input current at time *t*. The threshold-linear form is often chosen because of mathematical convenience, but also because it mimics to first order the gain function of many individual neurons in experiments (Chance et al. 2002; Blomquist et al. 2009), while the sigmoidal also models the saturation at very high firing rates. Yet, parameters of the gain function such as time constants, activation thresholds, or slope are often chosen rather qualitatively, and it is uncertain how well they match single-neuron properties or biophysics.

A first step towards a stringent comparison of spiking neuron network simulations with reduced neural mass or field models is to obtain an adequate quantitative expression for the neuronal gain function *F*(*I*(*t*)). It is hence of interest to understand if and how the activity of individual spiking neurons in response to arbitrary input currents can be described truthfully by a rate-model formulation. Several point neuron models are simple enough to allow for an analytical derivation of the gain function, assuming that input currents are Gaussian white noise, sinusoidally modulated input, or shot noise of a given structure (see, e.g., Gerstein and Mandelbrot 1964; Stein 1965; Brunel 2000; Brunel et al. 2001; Burkitt 2006b; Richardson 2007; Richardson and Swarbrick 2010; Roxin 2011; Ostojic and Brunel 2011). However, more complex nonlinear neuron models, such as the Izhikevich model or even the AMAT model, often render such analyses futile, especially in the presence of large-amplitude post-synaptic current events that are beyond the realm of perturbation-based theories. This holds to an even larger degree for the second step towards a stringent comparison of spiking network and neural field models, namely capturing the temporal response properties of the models. A thorough understanding of complex nonlinear models thus requires simulation studies.

We provide here an analysis of the response to spike train input of the models proposed by Izhikevich (2003b) and by Yamauchi et al. (2011), following the approach by Nordlie et al. (2010) and Heiberg et al. (2013). Both models actually represent an entire class of models that can be tuned to a wide range of reponses by adjusting model parameters. We will thus refer to the Izhikevich and AMAT *model classes*, respectively, when we refer to the set of equations and spike-generation rules, while we will call each of the approximately 20 different parameterization a *model*. Each of the two model classes comprises some 20 models.

In Section 3.1 we present how the different models respond to spike train input.

In Section 3.2, we present fits of a linear-nonlinear firing-rate model to the spike responses of Izhikevich and AMAT models to stationary and temporally modulated stochastic spike trains across a range of input rates, synaptic weights, and modulation frequencies and amplitudes under different background noise regimes.

We group the different models according to the filter parameters obtained in Section 3.3, before we in Section 3.4 explore how well the linear-nonlinear rate models capture the response of their spiking counterparts to novel stimuli, such as steps in the input firing rate and more complex temporally modulated input.

Finally, in Section 3.5 we investigate whether we can generalize models fitted to a specific input regime to a broader set of stimuli, before we summarize our findings in Section 4.

## Methods

### Neuron models

We study response behavior for two neuron model classes, the Izhikevich model (Izhikevich 2003b) and the augmented MAT model (Yamauchi et al. 2011). As both model classes are well described in the original publications, we just summarize them briefly in Tables 1 and 2. Both models are able to reproduce 20 of the most prominent features of biological spiking neurons in response to injected current input as illustrated in Fig. 1. These response types were first summarized in tabular form by Izhikevich (2004); see also Markram et al. (2004). Tables 3 and 4 present the parameter values required to obtain the model responses displayed for each class; note that certain models only differ in the stimulus injected, while neuron parameters are identical. All models are implemented on a fixed time grid (*dt* = 0.1ms).

**Table 1:**
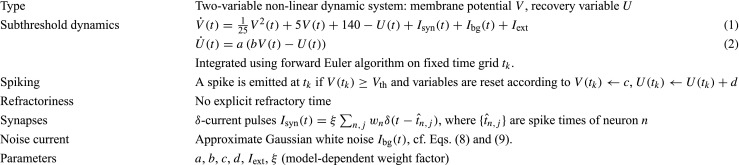
Summary of Izhikevich model; for parameters, see Table 3

**Table 2:**
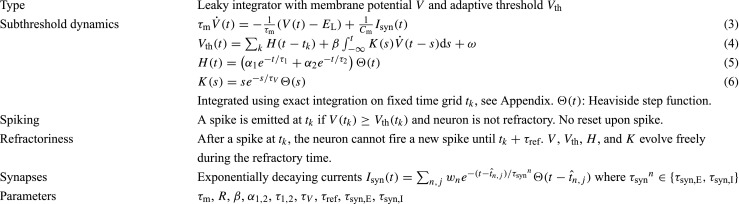
Summary of AMAT model; for parameters, see Table 4

**Table 3:** Parameters for Izhikevich model class obtained from code published by Izhikevich (2003a)

Label	Model	*a*	*b*	*c*	*d*	*ξ*	*I*_ext_
A	Tonic spiking	0.02	0.2	− 65	6	15.1	0
B	Phasic spiking	0.02	0.25	− 65	6	4.3	0
C	Tonic bursting	0.02	0.2	− 50	2	15.1	0
D	Phasic bursting	0.02	0.25	− 55	0.05	4.3	0
E	Mixed mode	0.02	0.2	− 55	4	15.1	0
F	Spike frequency adaptation	0.01	0.2	− 65	8	15.1	0
G*	Class 1 excitable	0.02	− 0.1	− 55	6	49	0
H	Class 2 excitable	0.2	0.26	− 65	0	5.6	− 0.5
I*	Spike latency	0.02	0.2	− 65	6	15.1	0
J	Subthreshold oscillation	0.05	0.26	− 60	0	1.8	0
K	Resonator	0.1	0.26	− 60	− 1	2.4	0
L*	Integrator	0.02	− 0.1	− 55	6	49	0
M	Rebound spike	0.03	0.25	− 60	4	4.5	0
N	Rebound burst	0.03	0.25	− 52	0	4.5	0
O*	Threshold variability	0.03	0.25	− 60	4	4.5	0
P	Bistability	0.1	0.26	− 60	0	0.87	0.24
Q	Depolarizing after-potential	1	0.2	− 60	− 21	17.8	0
R*	Accomodation	0.02	1	− 55	4	1	0
S	Inhibition-induced spiking	− 0.02	− 1	− 60	8	4.5	80
T*	Inhibition-induced bursting	− 0.026	− 1	− 45	− 2	4.8	80

Labels refer to subfigure labels in Izhikevich (2004, Fig. 1). Models A and I, G and L, and M and O, respectively, share the same parameters and differ only in their input parameters. Instances marked with an asterisk were not included in the study due to repeated parameters sets, non-standard model equations or pathological behavior; see text for details. Common parameters: *V*_th_ = 30mV, *V* (*t* = 0) ∼ *U*(− 70mV, 30mV)

**Table 4:** Parameters for AMAT model class, based on Yamauchi et al. (2011, Table 1)

Label	Model	*α*_1_	*α*_2_	*β*
A	Tonic spiking	10	0	0
B	Phasic spiking	10	0	− 0.3
C	Tonic bursting	− 0.5	0.35	0
D	Phasic bursting	− 0.5	0.35	− 0.3
E	Mixed mode	− 0.8	0.7	0
F	Spike frequency adaptation	10	1	0
G	Class 1 excitable	15	3	0
H	Class 2 excitable	15	− 0.05	0
I	Spike latency	10	0	− 1
J	Subthreshold oscillations	1	0	0.2
K	Resonator	10	0	0.5
L*	Integrator	10	0	0
M	Rebound spiking	10	0	− 2.5
N	Rebound bursting	− 0.5	0.35	− 2.5
O	Threshold variability	10	0	− 0.5
P	Bistability	20	− 0.4	0
Q	Depolarizing after-potential	25	− 1	0
R*	Accomodation	10	0	− 0.5
S	Inhibition-induced spiking	20	0	2
T	Inhibition-induced bursting	− 0.5	0.35	2

Note that models A and L and O and R, respectively, have identical parameters, whence L and R are not included in the study (marked with asterisk). Common parameters: *E*_L_ = − 70mV, *ω* = − 65mV, *C*_m_ = 200pF, *τ*_m_ = 10ms, *τ*_1_ = 10ms, *τ*_2_ = 200ms, *τ*_*V*_ = 5ms, *τ*_ref_ = 2ms, *τ*_syn,E_ = 1ms, *τ*_syn,I_ = 3ms, $V(t = 0)\sim U(-70 \text {mV}, -65 \text {mV})$. See text for difference between NEST parameterization and that in Yamauchi et al. (2011)

We integrate the Izhikevich model class using the forward Euler algorithm as in the original publications on the model. Izhikevich (2003b) used a 1 ms time step, but splitting the update of the membrane potential (but not the recovery variable) into two steps of 0.5 ms “for numerical stability”. Figure 1 of Izhikevich (2004), on the other hand, was generated using different time steps for different cases, ranging from 0.1 ms to 0.5 ms without substepping, as evidenced by the source code used to generate that figure (Izhikevich 2003a). We extracted model parameters as shown in Table 3 from that source code, including an external current *I*_ext_ injected into the model for some variants in addition to the stimulus current.

Izhikevich’s source code also revealed that model variants G, L, and R use other equations than Eqs. (??)–(??) for *V* (*t*) or *U*(*t*). We therefore excluded these variants from our study. We also excluded variants I and O, since they have the same parametes as variants A and M, respectively, and differ only in the test stimulus injected to create Fig. 1 of Izhikevich (2004).

Furthermore, we observed that response patterns depend on the precise time step used. In particular, the response for case T, *Inhibition-induced bursting*, is unstable for time steps shorter than 0.5 ms. We therefore also excluded case T from our analysis.

The Izhikevich model class is not defined with consistent units in the original publication (Izhikevich 2003b). While a time unit of milliseconds is implied and membrane potential is specified in millivolts, no units are given for the parameters or explicit constants. The model equations imply that input currents have units of mV/ms, which is rather exotic. In the spirit of Izhikevich (2003b) we therefore treat all quantities except time and membrane potential as unitless for the Izhikevich model class.

The AMAT class is implemented in NEST as model amat2_psc_exp using exact integration (Rotter and Diesmann 1999). The implementation follows the NEST convention of parameterizing the membrane potential equation Eq. (4) in terms of membrane time constant *τ*_m_ and membrane capacitance *C*_m_ and an explicit reversal potential *E*_L_, while Yamauchi et al. (2011) parameterize their Eq. 1 in terms of *τ*_m_ and membrane resistance *R* and define *E*_L_ = 0mV. The parameterizations are related by *C*_m_ = *τ*_m_/*R* and a shift of the membrane potential *V* and the resting value of the threshold *ω* by *E*_L_. Some parameter values were adjusted to be able to reproduce Figs. 6 and 7 in Yamauchi et al. (2011) as discussed in the Appendix. Model variants L and R are excluded from the study as they have identical parameters to variants A and O, respectively.

In all simulations reported here, a single neuron is stimulated with spike train input. For the Izhikevich model class, this spike input results in instantaneous jumps in the membrane potential *v*. For the AMAT class, each incoming spike evokes an exponentially decaying synaptic current. For details, see Tables 1 and 2 and Section 2.2.

Output spikes are recorded with NEST device spike_detector.

### Stimulation

We briefly summarize here the sinusoidal stimulation protocol and response characterization based on Nordlie et al. (2010) and presented in detail in Heiberg et al. (2013). More general stimulation protocols are described in Section 2.5.

**Fig. 2:**
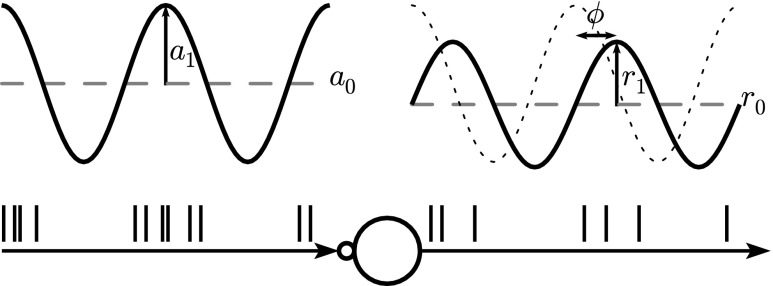
A model neuron is driven by a spike train with sinusoidally modulated rate *a*(*t*) with mean *a*_0_, modulation depth *a*_1_, and frequency *f*_stim_, cf. Eq. (7). As a first-order approximation, the output spike train of the neuron is characterized by the sinusoidally modulated response firing rate *r*(*t*) with mean *r*_0_, amplitude *r*_1_, frequency *f*_stim_ and phase *ϕ*, cf. Eq. (10). Adapted from Nordlie et al. (2010), Fig. 1

Model neurons are stimulated with sinusoidally modulated inhomogeneous Poisson process spike trains (Fig. 2) with rate (or intensity)

7 $$ a(t) = a_{0} + a_{1} \sin(2\pi{{f}_{\text{{stim}}}} t ) .  $$

Mean rates *a*_0_, modulation depth *a*_1_, and modulation frequency *f*_stim_ are varied systematically; modulation depth is limited to 0 ≤ *a*_1_ ≤ *a*_0_ to avoid rectification. We used NEST device sinusoidal_poisson_generator to generate the input spike trains.

The weights *w* > 0 of the synapses transmitting the stimulus spike train *a*(*t*) are varied from about 10*%* to about 75*%* of the synaptic weight *w*_*𝜃*_ required if a single incoming excitatory spike shall evoke a threshold crossing from rest. For the AMAT model class, *w*_*𝜃*_ is the same for all model variants and we use weights between 100 pA and 900 pA in our experiments.

For the Izhikevich model class, in contrast, model parameters do influence the response to isolated spikes. We therefore define a weight factor *ξ* for each model variant as the smallest weight for which a single excitatory input spike triggers the spike initiation process. Synaptic weights *w* are set to fractions of this value, ranging from 0.1 to 0.75, i.e., within the same range as for the AMAT model.

In addition to the resulting current stimulus, *I*_stim_(*t*), we consider stationary noisy background input currents *I*_bg_(*t*), representing unspecific weak network input. This allows us to study neuronal responses to *I*_stim_(*t*) in different input scenarios. The full input a neuron receives is thus given by *I*(*t*) = *I*_stim_(*t*) + *I*_bg_(*t*). We characterize the background current by its mean *μ*_bg_ and standard deviation *σ*_bg_.

The NEST implementation of the Izhikevich neuron model is equipped with instantaneous current-based synapses. Assuming high rates and small synaptic strength, balanced spiking input can be approximated well by Gaussian white noise. We thus inject approximate Gaussian white noise realizations of defined mean *μ*_bg_ and standard deviation *σ*_bg_ using NEST’s noise_generator^2^.

The AMAT model, as used here, has current-based exponential synapses with characteristic time constants *τ*_syn,E_ = 1ms and *τ*_syn,I_ = 3ms. We inject background current as Poisson spike trains through synapses with small, fixed weight *w*_E,bg_ = 1pA and *w*_I,bg_ = − 4/3 pA, respectively, using NEST model poisson_generator. The resulting noise input current has mean and standard deviation

8 $$\begin{array}{@{}rcl@{}} \mu_{\text{bg}}&=&w_{\text{E,bg}}\nu_{\text{E}}\tau_{\text{syn,E}}+w_{\text{I,bg}}\nu_{\text{I}}\tau_{\text{syn,I}} \end{array} $$

9 $$\begin{array}{@{}rcl@{}} \sigma_{\text{bg}}&=&\sqrt{w^{2}_{\text{E,bg}}\nu_{\text{E}}\tau_{\text{syn,E}}/2+w^{2}_{\text{I,bg}}\nu_{\text{I}}\tau_{\text{syn,I}}/2} . \end{array} $$

For given *μ*_bg_ and *σ*_bg_, we obtain noise input rates by solving Eqs. (8)–(9) for *ν*_E_ and *ν*_I_.

We consider three background current regimes: first the case without additional background current *I*_bg_(*t*) = 0pA, where all spiking activity is purely stimulus induced. In the second case, *I*_bg_(*t*) is chosen such that *μ*_bg_ = 0pA, and *σ*_bg_ is large enough to elicit spiking activity with background input alone, i.e., if *I*_stim_(*t*) = 0pA. In the third case, we consider a net inhibitory background current, with *μ*_bg_ < 0pA and sufficient standard deviation *σ*_bg_ to again elicit baseline spiking in absence of *I*_stim_(*t*). While the first scenario can be considered a typical situation for neurons in slice preparations, the latter two mimic the situation *in vivo*, e.g., in cortical layer II/III where ongoing spiking activity is sparse (see e.g., Sakata and Harris 2012; Petersen and Crochet 2013) and input currents are balanced or even inhibition dominated (Haider et al. 2013).

### Characterization of response properties

#### Sinusoidal rate model

We characterize the response of the neurons by a sinusoidal rate model

10 $$\begin{array}{@{}rcl@{}} r(t) &=& r_{0} + r_{1} \cos(2\pi {{f}_{\text{{stim}}}} t + \phi_{1})\\ &&+ \sum\limits_{m = 2}^{\infty} r_{m} \cos(2 m \pi {{f}_{\text{{stim}}}} t + \phi_{m}) , \end{array} $$

as illustrated in Fig. 2. For a purely linear response, *r*_0_ represents the background firing rate of the neuron, *r*_1_ the stimulus response amplitude (with phase shift *ϕ*_1_), and we expect *r*_*m*_ = 0 for all higher harmonics (*m* ≥ 2). Any nonlinearities in the system will typically be associated with power in the higher harmonics. We consider power at harmonics as significant (*z*-test, 99% confidence level) if

11 $$ r_{m} > r_{\text{crit}} = B + 2.34{\Sigma} , $$

where *B* is the estimated background power of the spike train between the harmonics and Σ the weighted standard deviation of the spike train power spectrum across frequencies. For details, see Section 2.2.2 of Heiberg et al. (2013).

#### Linearity

We proceed as follows to characterize the linearity of the firing-rate curve in response to stationary input: We obtain the firing-rate curve *r*_0_ = *f*(*a*_0_) for a given neuron model, noise regime and synaptic weight by measuring the output rate *r*_0_ as a function of stationary input rate *a*_0_ in the absence of modulation (*a*_1_ = 0). To characterize the linearity of *f* over an interval [*α*,*β*], we define the linearity measure

12 $$ \bar{L}_{1} = \frac{{\int}_{\alpha}^{\beta} \left[ f(x) - \ell(x)\right]^{2} dx}{\beta-\alpha} \left/ \ell^{2}\left( \frac{\alpha+\beta}{2}\right)\right. ,  $$

as the normalized mean square difference between *f*(*x*) and *ℓ*(*x*), the best linear fit to *f*(*x*) over [*α*,*β*]. If *f*(*x*) is perfectly linear, we have $\bar {L}_{1}= 0$, while $\bar {L}_{1}= 1$ means that the average squared distance between firing-rate curve and linear fit is equal to the mean value over the interval. Larger values of $\bar {L}_{1}$ are difficult to interpret, though. We therefore define

13 $$ L_{1} = \frac{1}{1+\bar{L}_{1}} $$

as linearity measure. *L*_1_ = 1 indicates perfect linearity, *L*_1_ = 1/2 a deviation from linearity equal to the mean value, while *L*_1_ approaches 0 for large deviations. For a given mean rate *a*_0_ and modulation depth *a*_1_, we evaluate linearity over [*α*,*β*] = [*a*_0_ − *a*_1_,*a*_0_ + *a*_1_], i.e., the range of rates spanned by the temporally modulated input.

### Rate model description

The response of a linear, time-invariant (LTI) system to any input can be calculated as a convolution of the input and the *impulse response* of the system. A wide class of non-linear systems can be described by a linear convolution of the input with a kernel *h*(*t*) followed by a non-linear *activation function* *g*(⋅), so that the response is given by

14 $$ r(t) = g(h(t)*a(t)) . $$

To test how well this applies to the neuron models studied here, we fit linear-nonlinear firing-rate models to the responses of the spiking neuron models and compare firing-rate predictions from the linear-nonlinear models to those of the fitted spiking models. We summarize the derivation of the firing-rate model below, based on Heiberg et al. (2013) and Nordlie et al. (2010).

For each neuron, we find the activation function *g*(⋅) and the kernel *h*(*t*). For constant input, *a*(*t*) = *a*_0_, the convolution becomes the identity operation, provided the kernel is normalized ($\int h(t) dt= 1$). We determine *g*(⋅) by measuring the response to stationary input, *r*_0_ = *g*(*a*_0_) for a range of *a*_0_ and obtain a continuous representation of *g*(⋅) by interpolation (linear B-spline).

To obtain the kernel *h*(*t*), we linearize the activation function around a given *working point*(*a*_0_,*r*_0_). The response to *a*(*t*) = *a*_0_ + *a*_1_*s*(*t*) can then be expressed as

15 $$ \begin{array}{llllll} r(t) &= g(h(t)*(a_{0}+a_{1} s(t)) )\\ &\approx r_{0}(a_{0}) + h_{0}(t; a_{0}, a_{1}) * (a_{1} s(t)) , \end{array} $$

where the linear impulse response function

16 $$ h_{0}(t; a_{0}, a_{1}) = g^{\prime}(a_{0})h(t; a_{0}, a_{1})=:\gamma h(t; a_{0}, a_{1}) $$

combines the normalized kernel with the linear gain *γ*. In general, this approximation is only valid for small deviations from the working point. However, the limits are not known a priori. For brevity of notation, we will usually drop the explicit reference to stimulus parameters *a*_0_ and *a*_1_ below.

We obtain the transfer function, i.e., the Fourier transform of the linear impulse response *h*_0_(*t*), from the model responses to sinusoidally modulated input (*s*(*t*) = sin2*πf*_stim_*t*, cf. Eq. (7))

17 $$ H_{0}({{f}_{\text{{stim}}}}) = \frac{r_{1}({{f}_{\text{{stim}}}})}{a_{1}}e^{i\phi({{f}_{\text{{stim}}}})} $$

where *r*_1_(*f*_stim_) and *ϕ*(*f*_stim_) are the Fourier amplitude and phase of the response, respectively.

In Nordlie et al. (2010) and Heiberg et al. (2013), first-order low-pass filters with delay provided adequate fits to the empirical frequency responses. Here, more complex filter models are needed to fit additional response features. In particular, we expect a second filter time constant *τ*_c_ = 1/(2*πf*_c_) to be needed to model some of the response types illustrated in Fig. 1. We choose to combine low- and high-pass components of the filter as a sum:

18 $$ \tilde{H}_{0,\text{SUM}}(f) = \gamma_{1} e^{-2\pi if{\Delta}} \left( \frac{1}{1+ i\frac{f}{f_{\text{c,1}}}} + \frac{\gamma_{2}}{1+ i\frac{f}{f_{\text{c,2}}}} \right) . $$

This form allows for a representation of the filter through a system of linear differential equations, see Section 2.4.1. 3

The filter kernels were fitted to the empirical transfer function to capture it with as few parameters as possible. For each set of stimulus parameters (*a*_0_, *a*_1_, *w*, *μ*, *σ*), we obtained fits for the parameters *γ*_1_, *f*_c,1_, *γ*_2_, *f*_c,2_ and Δ. Fitting was performed using basin-hopping optimization provided by the SciPy Optimize toolbox with L-BFGS-B minimization (Jones et al. 2001). To avoid pathological solutions, we imposed the following constraints

19 $$\begin{array}{@{}rcl@{}} 0.25 \text{ms} \leq \tau_{\text{c},1,2} \leq 175 \text{ms} \end{array} $$

20 $$\begin{array}{@{}rcl@{}} \quad \Leftrightarrow \quad 0.909 \text{Hz} \leq f_{\text{c},1,2} \leq 636.6 \text{Hz} \end{array} $$

21 $$\begin{array}{@{}rcl@{}} 0 \leq {\Delta} \leq 75 \text{ms} . \end{array} $$

If a fit resulted in *f*_c,1_ > *f*_c,2_, we swapped frequencies and gain coefficients

22 $$\begin{array}{@{}rcl@{}} f_{\text{c},1}, f_{\text{c},2} &\leftarrow f_{\text{c},2}, f_{\text{c},1} \end{array} $$

23 $$\begin{array}{@{}rcl@{}} \gamma_{1}, \gamma_{2} &\leftarrow \gamma_{1}\gamma_{2}, 1/\gamma_{2} \end{array} $$

so that *f*_c,1_ and *f*_c,2_, respectively, are always the lower and upper characteristic frequencies of the filter. For each parameter combination, we performed 60 independent fits from different starting points and retained the best fit. We also performed 15 independent fits for a pure lowpass filter, but these never yielded better results than fits to the bandpass filter defined by Eq. (18).

We now define the linear-nonlinear rate model as

24 $$ {r_{\text{NL}}}(t) = \max(0, g(h(t) * a(t))) , $$

with the normalized kernel

25 $$ h(t) = \frac{h_{0}(t)}{\gamma_{1}(1+\gamma_{2})} $$

and correspondingly in Fourier space. We take the maximum solely to avoid negative rates that may result in rare cases from extrapolation of the activation function *g*(⋅). This kernel depends on the stimulus parameters *a*_0_ and *a*_1_ used to construct it, but we drop this dependence for clarity of notation; the actual range over which a kernel is useful is explored in Section 3.

#### Differential-equation representation

The filter $\tilde {H}_{0,\text {SUM}}(f)$ corresponds to a sum of low-pass filters in the time domain. For this model, the linear-nonlinear model of Eq. (14) can be mapped to a set of delay differential equations using the linear chain trick (Nordbø et al. 2007).

In particular, in the time domain the filter is given by

26 $$\begin{array}{@{}rcl@{}} h_{0,\text{SUM}}(t + d) = h_{0,1}(t +d) + h_{0,2}(t +d)\\ = {\Theta}(t) \left( \frac{\gamma_{1}}{\tau_{1}}e^{-t/\tau_{1}}+\frac{\gamma_{1} \gamma_{2}}{\tau_{2}}e^{-t/\tau_{2}}\right) \end{array} $$

with Heaviside step function Θ(*t*). We introduce

27 $$ u(t) = (a*h_{0,\text{SUM}})(t)=u_{1}(t)+u_{2}(t) $$

with

28 $$ u_{1}(t) = (a*h_{0,1})(t)\quad\text{and} \quad u_{2}(t) = (a*h_{0,2})(t) . $$

Straightforward differentiation of the two temporal kernels yields

29 $$\begin{array}{@{}rcl@{}} \dot{u_{1}}(t) &=& -\frac{u_{1}(t)}{\tau_{1}}+ a(t)\frac{\gamma_{1}}{\tau_{1}} \end{array} $$

30 $$\begin{array}{@{}rcl@{}} \text{and}\quad \dot{u_{2}}(t) &=&-\frac{u_{2}(t)}{\tau_{2}} + a(t)\frac{\gamma_{1}\gamma_{2}}{\tau_{2}} . \end{array} $$

From this we can solve for *u*(*t*) = *u*_1_(*t*) + *u*_2_(*t*) and the full rate dynamics then follows by application of the nonlinearity *g*

31 $$ r(t) = g\left( u(t)\right) . $$

The advantage of the differential representation Eq. (29) lies in the fact that it is local in time, whereas the representation by convolution in general relies on knowledge of the complete history of the dynamics.

### Tests against spike trains

We compare the response properties of our rate-based model against spiking neuron models as follows. We use synthetic (Section 2.5.1) or experimentally recorded (Section 2.5.2) spike trains *S*(*t*) as test input. Spiking neuron models are driven by these trains directly and their output spike trains *R*(*t*) are recorded as described in Section 2.6. We then use the fixed-kernel density estimation method by Shimazaki and Shinomoto (2010) with 0.05 ms bin width to estimate a continuous output firing rate *r*_spike_(*t*). This is the reference against which we test the rate-based model.

To obtain the response of the rate-based model, we either use the known rate of the synthetic input spike trains or obtain a continuous input rate function *a*(*t*) from the input spike trains *S*(*t*) using the fixed-kernel density estimation method. Applying Eq. (14) to this rate yields the reponse of the rate model *r*_rate_(*t*).

We repeat each simulation experiment with five different random seeds and retain only results for which the optimal kernel width obtained by the densitiy estimation methods is 15 ms or less, as wider kernels would lead to an undue smoothing over time.

The difference between responses obtained from rate-based and spiking models is then defined as the mean squared error normalized by the variance of the response of the spiking model (Pillow et al. 2005)

32 $$ \bar{E}_{r} = \frac{ {{\int}_{0}^{T}}\left( {{r}_{\text{{rate}}}}(t)-{{r}_{\text{{spike}}}}(t)\right)^{2} \text{d}t} {{{\int}_{0}^{T}}\left( {{r}_{\text{{spike}}}}(t)-{{\bar{r}}_{\text{{spike}}}}\right)^{2}\text{d}t} $$

where ${{\bar {r}}_{\text {{spike}}}}$ is the average response rate of the spiking model. Corresponding to the linearity measure *L*_1_ (Section 2.3.2), we define

33 $$ E_{r} = \frac{1}{1+\bar{E}_{r}} $$

as quality measure. *E*_*r*_ = 1 indicates perfect agreement, *E*_*r*_ = 1/2 an error equal to the variance, while *E*_*r*_ approaches 0 for large deviations between spiking and rate model response.

#### Tests with synthetic spike trains

We first test models using a Poisson spike train input with step changes in rates. Stimulus parameters are given in Table 5. Spike responses are obtained by simulating a population of 4,096 independent model neurons driven by one Poisson process each. The resulting 4,096 output spike trains are pooled to estimate the output rate *r*_spike_(*t*).

**Table 5:** Poisson spike train rates applied during different intervals

Interval [ms]	0–600	600–1000	1000–1200	1200–1500
Rate [1/s]	100	200	40	150

Rates change instantaneously at interval boundaries

#### Tests with realistic spike trains

**Fig. 3:**
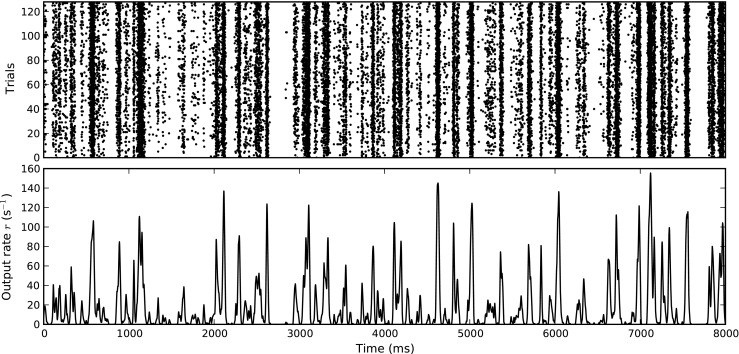
Spike raster and rate profile for retinal ganglion cell (RGC) data used to test model performance. Rates were estimated by means of kernel density estimation (Shimazaki and Shinomoto 2010), using the *fixed kernel* method

To test the performance of the rate models in response to realistic spike trains, we drive model neurons by spike trains recorded from retinal ganglion cells (RGCs) in cat (Casti et al. 2008). Their data set contained 128 spike trains of 8 s duration recorded during different trials, characterized by low baseline firing and fast transients as illustrated in Fig. 3. Because trains from the first trials in the dataset have noticeably lower average firing rates than those from later trials, we only use the last 96 spike trains with an average firing rate of 18.3 ± 1.3 spikes per second.

The Izhikevich models in particular responds weakly to these spike trains in many cases. We therefore increase the rate of the input spike trains by merging pairs of spike trains, resulting in a total of 48 input spike trains with average rates of 36.6 spikes per second. We then drive 48 model neurons independently with one spike train each for 8000 ms and pool the resulting output spike trains for output rate estimation.

### Simulation

Simulations for all model configurations are performed with the NEST Simulator (Gewaltig and Diesmann 2007; Plesser et al. 2013).

In practice, we simulate *N* trials by creating *N* mutually independent Poisson-generator–neuron pairs in a single NEST simulation. Membrane potentials are randomized upon network initialization and data collection is started only after an equilibration period of 1 s simulated time. All simulations are performed with a spike-time resolution of 0.1 ms.

Simulations underlying model fitting are performed using NEST 2.3.r10450, while some scoring of model responses according to Eq. (33) was performed using NEST 2.8.0. Trials are configured using the NeuroTools.parameters package (Muller et al. 2009). Data analysis is performed using NumPy 1.7.1–1.11.1, SciPy 0.18.1, Pandas 0.11.0–0.18.1 and Matplotlib 1.2.1–1.5.3 under Python 2.7.

## Results

### Response to spike train input

**Fig. 4:**
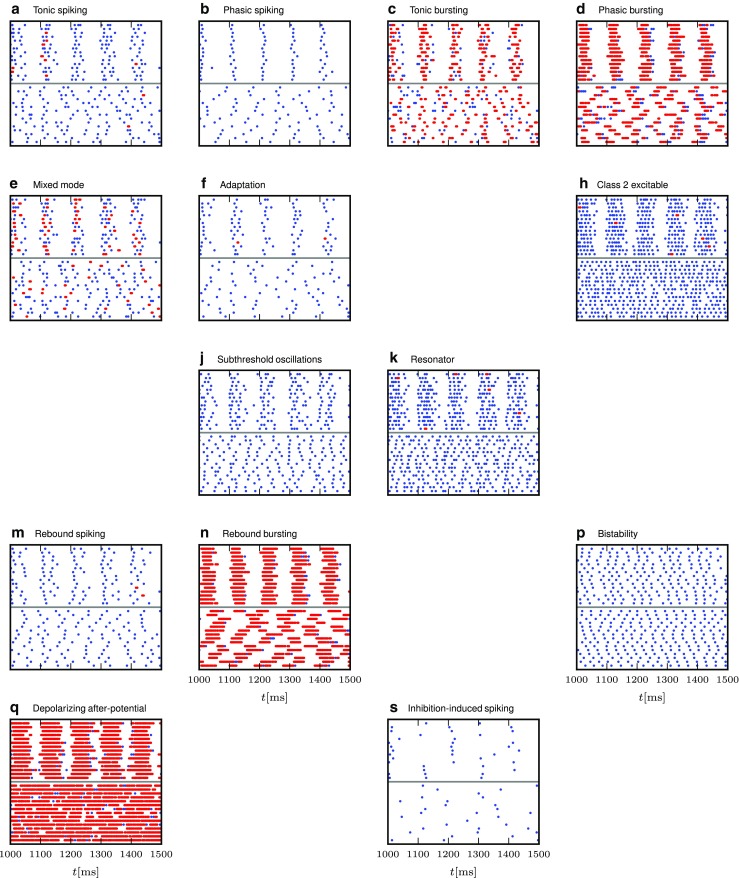
Spike responses of Izhikevich models driven by stationary (rate $a_{0}= 400 \text {s}^{-1}$) or sinusoidally modulated (rate $a_{0}= 400 \text {s}^{-1}$, modulation amplitude $a_{1}= 400 \text {s}^{-1}$, modulation frequency 10Hz) Poisson spike trains impinging with synaptic weight *w* = 0.6pA weighted with *ξ*, and noise (*μ* = 0pA, *σ* = 0.1pA). The upper part of each panel shows the response of 15 neurons driven by different sinusoidally modulated spike trains, the lower part the response of 15 neurons driven by different stationary trains after 1 s of equilibration time. Isolated spikes are shown in blue, clustered spikes within *dT* = 5ms from another spike in red

**Fig. 5:**
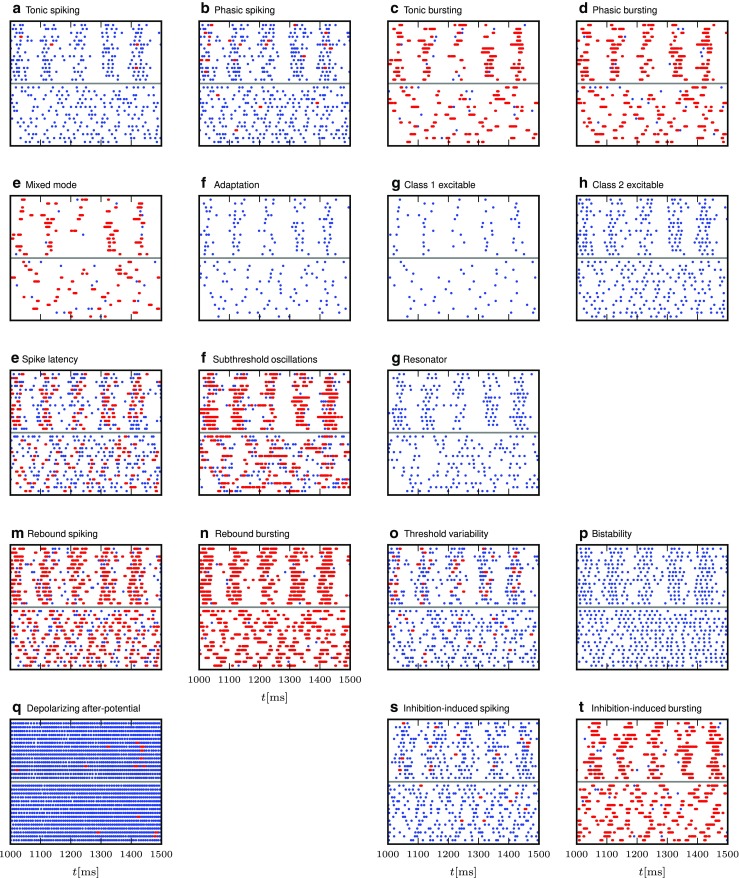
Spike responses as for AMAT models as in in Fig. 4, using weight *w* = 200pA, and noise with *μ* = 0pA, and *σ* = 100pA

To gain a first impression of the basic response properties of the models, we show the spike responses to stationary and sinusoidally modulated Poisson input in Figs. 4 and 5 for Izhikevich and AMAT models, respectively. Each raster plot shows the response of 30 unconnected neurons, half driven by stationary and half by sinusoidally modulated Poisson spike trains after an equilibration phase of 1000 ms. Each of the 30 neurons receives different realizations of input spike trains and noise, but the same trains and noise are used for all models.

As spiking and bursting variations are included as separate response types in the model classification (Fig. 1), we illustrate the burstiness of the responses by marking spikes fired within *dT* = 5 ms of each other as belonging to a burst, corresponding to the upper limit of intra-burst intervals in LGN (Funke and Wörgötter 1997, p. 71).

The models that exhibit their characteristic behaviour (Fig. 1) based on “simple” excitatory input current shapes (e.g., steps, ramps, pulses) generally behave as expected when driven by Poisson spike trains; spiking neurons primarily spike and bursting neurons burst, but the nuances of individual models are less visible in the spiking patterns (e.g. tonic vs phasic) due to the input variability. Models that are based on more specific input current pattens or consistent inhibitory input (i.e., bottom rows) do to a lesser extent receive the required input and respond in a less characteristic manner, some even seem erratic (e.g., Fig. 5Q). Note, however, that the figures illustrate responses at a single input rate and noise regime combination and that the models to varying degree are sensitive to these conditions.

In contrast to the 20 markedly different responses to current injections (Fig. 1), responses to spiking input show more similar patterns across models, differing in the overall response rate and the proportion of spikes belonging to bursts.

While some Izhikevich and AMAT models that show identical responses to current injections also respond similarly when driven by spiking input (e.g., top two rows), we observe some with very different response patterns (e.g., depolarizing after-potential (Q) and inhibition-induced spiking (S)) across the two model classes.

### Linear-nonlinear models

We now obtain the linear-nonlinear models as defined by Eq. (24).

#### Activation functions

We first obtain the activation function *g*(⋅) by fitting a linear B-spline to the response to stationary input, *r*_0_ = *g*(*a*_0_), varying *a*_0_ from 0s^− 1^ to 1000s^− 1^ in steps of 10s^− 1^. This yields one activation function fit for

- each model (14 models for the Izhikevich model class, 18 for the AMAT model class);
- each background noise regime
  no noise: *μ* = 0,*σ* = 0
  balanced noise: Izhikevich: *μ* = 0,*σ* = 0.1, AMAT: *μ* = 0pA,*σ* = 100pA
  biased noise: Izhikevich: *μ* = − 0.1,*σ* = 0.2, AMAT: *μ* = − 100pA,*σ* = 200pA;
- each synaptic weight (Izhikevich: 0.1, 0.25, 0.5, 0.6, 0.75; AMAT 100pA, 300pA, 500pA, 700pA, 900pA).

We thus obtain a total of 210 activation functions for the Izhikevich model class and 270 for the AMAT model class.

**Fig. 6:**
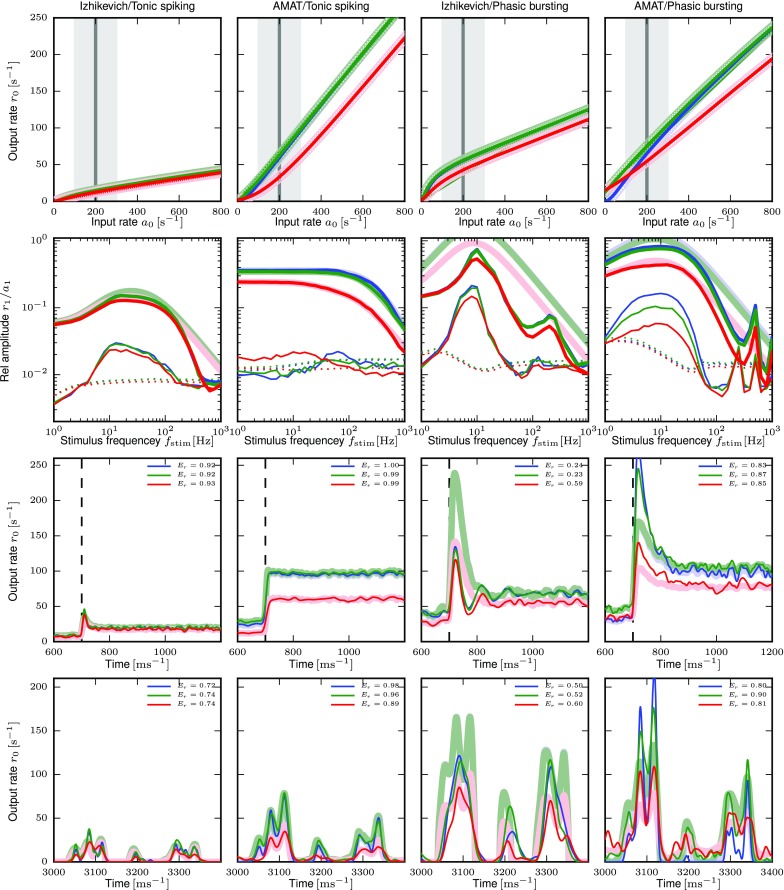
Response properties for exemplary model neurons. Columns show from left to right response properties of Izhikevich and AMAT tonic spiking and Izhikevich and AMAT phasic bursting models. Top row: stationary output firing rate response *r*_0_ as function of input rate *a*_0_ for three noise regime levels (blue: no noise, green: balanced noise, red: biased noise). Light symbols show responses from simulations, solid lines the fitted B-splines. Second row: Frequency response to sinusoidally modulated Poisson input with mean $a_{0}= 200~\text {s}^{-1}$ and modulation amplitude $a_{1}= 100~\text {s}^{-1}$ as function of modulation frequency *f*_stim_; thick solid lines: first harmonic *r*_1_, thick shaded lines: fitted filter *H*_0_(*f*), thin solid lines: second harmonic *r*_2_, dotted lines: significance level *r*_crit_. Mean input rate *a*_0_ and modulation range *a*_0_ ± *a*_1_ are marked gray in the top row. Fit parameters are given in Table 6. Third row: Response of spiking model (thin solid lines) and rate-model prediction (light thick lines) to Poisson spike trains with rate 100 s^− 1^ for *t* < 700 ms and 300 s^− 1^ for *t* ≥ 700 ms. Fit quality *E*_*r*_ shown as inset. Bottom row: Response to realistic spike trains, 400 ms section starting at 3000 ms (cf. Fig. 3) with the same line types as for step responses. Connection weight *w* from left to right: 0.75, 700, 0.75, 500

The top row of Fig. 6 shows the activation function for the Tonic Spiking and the Phasic Bursting models for the Izhikevich and AMAT model classes, respectively, for all three noise regimes. The spike rates obtained from the simulations are fitted very well by the B-splines. This holds for all models except model S in the Izhikevich class (inhibition-induced spiking), which has rather noisy activation curves with extremely high rates under certain conditions (above 1000s^− 1^); data not shown.

**Fig. 7:**
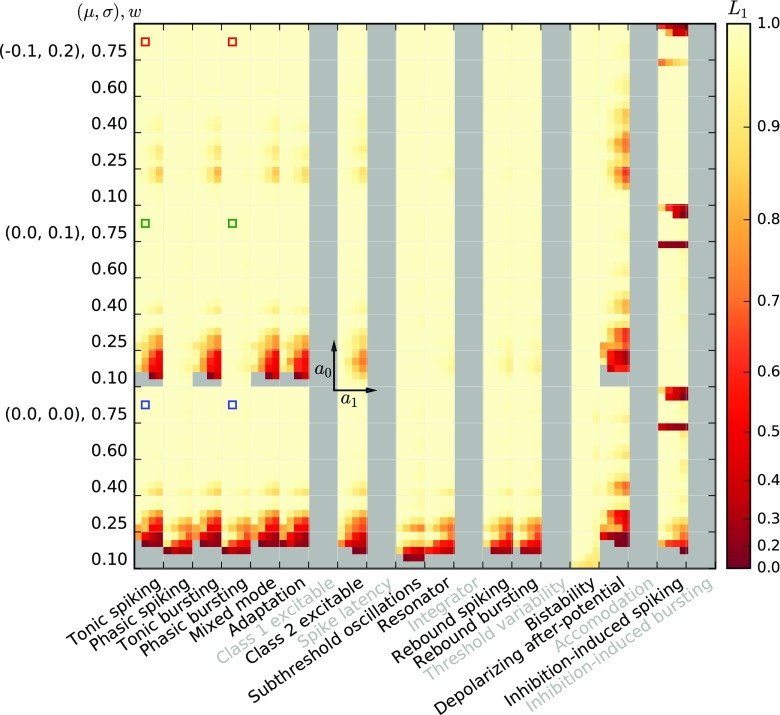
Linearity measure *L*_1_ for the Izhikevich model class for different model parameterizations (major columns), and five different synaptic weights for each of three noise regimes (major rows). Each major row/column block shows data for five different average input rates ($a_{0}=\{50, 100, 200, 400, 800\} \text {s}^{-1})$, minor rows) and four different modulation amplitudes (*a*_1_ = {0.25,0.5,0.75,1.0}× *a*_0_, minor columns), as indicated by the small coordinate axes shown for one major square. Thus, the bottom left minor square of each major square is the *L*_1_ value computed over 37.5s^− 1^ ≤ *a* ≤ 62.5s^− 1^, while the upper right minor square is the *L*_1_ value computed over 0s^− 1^ ≤ *a* ≤ 1600s^− 1^. *L*_1_ = 1 indicates perfect linearity of the firing-rate curve *F*(*a*) over the relevant input range, cf. Section 2.3.2. Grey indicates missing data, either because neurons were unresponsive or because the model variants are described by non-standard differential equations or have duplicate parameters. Blue, green, and red squares correspond to the examples shown in the first and third columns of Fig. 6

**Fig. 8:**
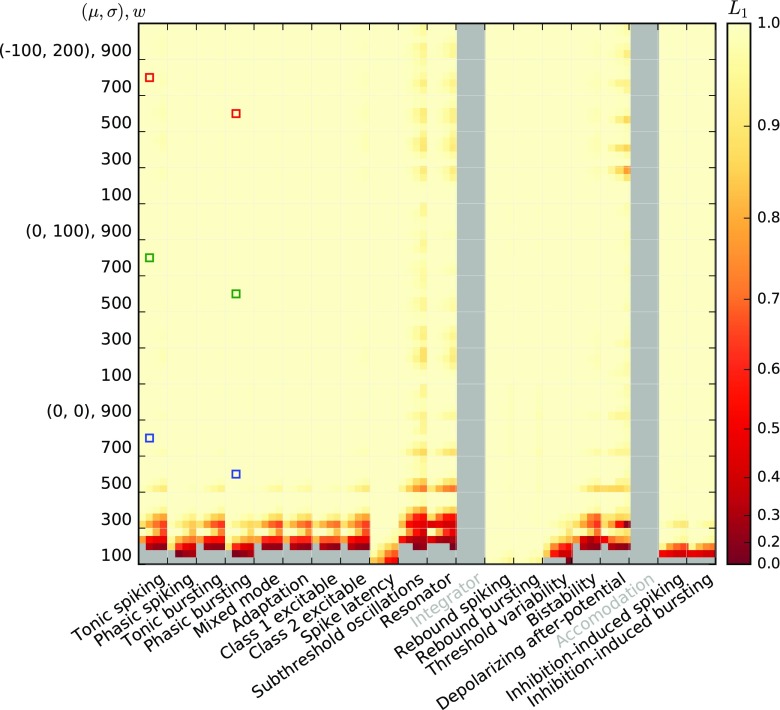
Linearity measure *L*_1_ for the AMAT model; the figure is constructed as Fig. 7

Before we investigate the response of the models to temporally modulated stimuli, we briefly explore the linearity of the activation functions around different working points *a*_0_ = {50s^− 1^,100s^− 1^,200s^− 1^,400s^− 1^,800s^− 1^} and modulation amplitude *a*_1_ = {0.25,0.5,0.75,1}× *a*_0_ about the working point. A working point at *a*_0_ = 200s^− 1^ with modulation amplitude *a*_1_ = 100s^− 1^ is shaded in the top row of Fig. 6 for illustration. Figures 7 and 8 show the linearity score *L*_1_ for each of the 20 (*a*_0_, *a*_1_) combinations for each response curve.

For the Izhikevich neurons, the stationary linearity metric *L*_1_ indicates that strong synaptic weights *w*, large mean input rates *a*_0_, and small modulation amplitudes *a*_1_ give the most linear responses. Larger weights and mean rates not only increase the mean input of the Poisson input current, but also its variance. This leads to a linearization of the activation function and moves the activation threshold towards smaller rates (see also Chance et al. 2002). Furthermore, firing-rate modulation amplitudes are more likely to stay within a single region of the sigmoidal firing rate curve for small *a*_1_, and are thus more likely to adhere to a linear fit.

The stationary linearity metric *L*_1_ for the augmented MAT model indicates overall more linear behavior, but the same general pattern of parameter dependence can be seen (Fig. 8). One notable difference is the saturation of the AMAT model at output rates of 500 s^− 1^—due to the absolute refractory time of 2 ms—that adds another source of non-linearity in the firing rate curves for some neurons.

#### Transfer function and linear filters

We obtain empirical transfer functions according to Eq. (17) for 20 combinations of working point and modulation depth (*a*_0_,*a*_1_) for each model, noise regime and synaptic weight using the approach described in detail in Heiberg et al. (2013, Section 2.2.2), measuring the model response at 28 different stimulation frequencies *f*_stim_, logarithmically spaced from 1Hz to 1000Hz. We then fit the linear filter $\tilde {H}_{0,\text {SUM}}(f) $ according to Eq. (18) as described in Section 2.4, obtaining fit parameters (*f*_*c*,1_,*f*_*c*,2_,*γ*_1_,*γ*_2_,Δ) for each model and stimulation parameter combination. Note that *γ*_1_ is fully captured by the activation function, and therefore does not explicitly enter the linear-nonlinear model we construct here, cf. Eq. (25).

The second row of Fig. 6 shows the resulting transfer functions and fitted kernels for the same models and conditions as the activation functions discussed above; see Table 6 for fit parameters. The examples reveal bandpass behaviour for three out of four cases, which also show significant power in the second harmonic *r*_2_. The exception is Tonic spiking for the AMAT class, which shows lowpass behavior and no significant power in *r*_2_. Phasic bursting shows a second peak in the spectrum around 200 Hz (Izhikevich) and 500 Hz (AMAT), which our fitted bandpass filter models (thick light lines) cannot capture by construction. These peaks occur as refractory effects regularize firing patterns at high rates. The fitted bandpass filters capture the frequency response of the model neurons well, except for Izhikevich phasic bursting case, where the amplitude of the fitted filter is significantly larger than the power in the first harmonic.

**Table 6:** Fit parameters for filters *H*_0_(*f*) shown in the second row of Fig. 6

Model	Noise	*γ*_1_	*γ*_2_	*f*_c,1_[Hz]	*f*_c,2_[Hz]	Δ[ms]
Izh/Tonic spiking	none	− 0.152	− 1.328	9.988	61.577	0.987
	balanced	− 0.150	− 1.334	9.959	63.535	1.009
	biased	− 0.110	− 1.442	8.397	68.001	1.003
AMAT/Tonic spiking	none	0.468	− 0.225	224.270	636.620	0.183
	balanced	0.386	− 0.107	181.140	636.620	0.181
	biased	0.088	1.748	28.946	149.427	0.222
Izh/Phasic bursting	none	− 25.548	− 1.002	8.745	9.043	1.709
	balanced	− 23.825	− 1.002	8.817	9.134	1.670
	biased	− 21.662	− 1.003	8.601	8.892	1.936
AMAT/Phasic bursting	none	− 0.718	− 1.486	3.067	22.380	0.913
	balanced	− 0.672	− 1.488	3.159	21.450	0.832
	biased	− 0.304	− 1.884	3.697	21.139	0.836

We found that not all model variants responded sufficiently to periodic stimulation under all stimulation conditions to provide sufficient spike data to fit a kernel. Therefore, we only obtained kernel fits for approximately three-quarters of all conditions for the Izhikevich class (3262 out of 4200 possible) and about 90% of all conditions for the AMAT class (4843 out of 5400).

### Grouping of models

**Fig. 9:**
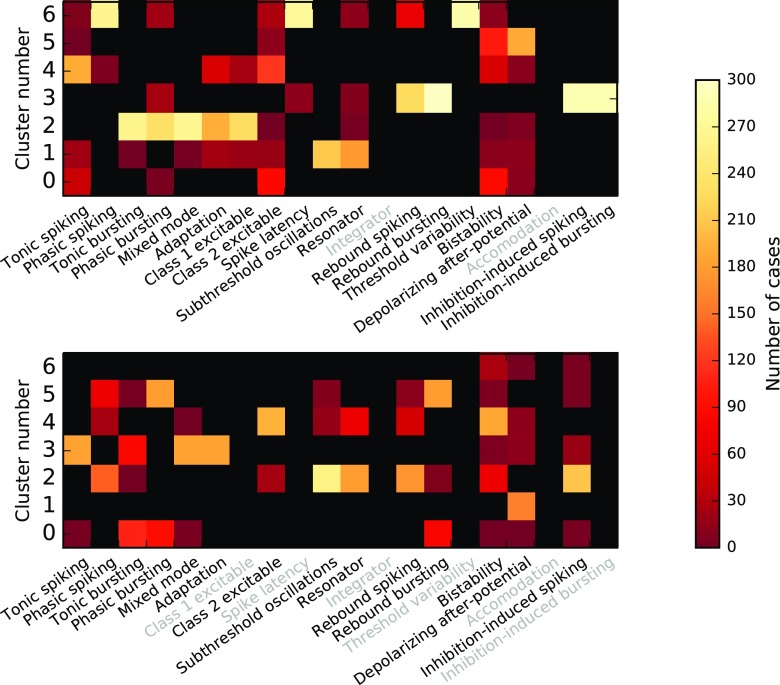
Result of *k*-means clustering of linear filter fit parameters for the AMAT (top) and Izhikevich (bottom) model classes. Rows represent clusters, columns model variants and the color of each square indicates how many stimulation configurations were assigned to a given cluster for of each model variant. Each model variant is assigned to the cluster for which it reached the highest count of stimulation configurations. Black means zero count, and models not considered are indicated by greyed-out labels

To systematize model responses, we cluster the kernel fit parameter sets^4^ with *k*-means clustering using Scikit-Learn (Pedregosa et al. 2011). We cluster Izhikevich and AMAT filters independent of each other. To find a suitable clustering, we ran the clustering algorithm searching for four, five, six, and seven clusters. For each run, we clustered starting from 100 initial conditions to avoid local minima. Since we are clustering fit parameters obtained for a wide range of simulation conditions, while we are interested in grouping the 14 and 18 model variants, respectively, we assign each model variant to a *model group* as follows: We count how often each model occurs in each *k*-means cluster and assign each model to the cluster to which it is assigned most often. Each cluster to which at least one model is thus assigned forms a model group. Low number of clusters led to model groups containing models with clearly different spiking behavior, e.g., for the AMAT model, the strongly bursting case J would be grouped with cases A, H and K, which show almost no bursts at all, jf Fig. 5 when using six clusters. We thus used seven clusters in all cases which yielded six model groups. Figure 9 shows the counts for each model in each cluster, with a maximum possible count of 300 if fit parameters are available for all combinations of *μ*,*σ*,*w*,*a*_0_,*a*_1_ and are assigned to the same cluster.

The resulting grouping into six groups per model class is shown in Table 7, with median kernel parameter values for each group in Table 8. Grouping is clearly different for the Izhikevich and AMAT classes, supporting our previous observation that these models respond differently to spike input even though they show identical responses to the current injection protocol of Fig. 1.

**Table 7:** Models grouped by *k*-means clustering of linear filter parameters as illustrated in Fig. 9

Group	Firing pattern	Izhikevich	AMAT
1	Isolated spikes, rare mini bursts	A/Tonic spiking	B/Phasic spiking
		E/Mixed mode	I/Latency
		F/Adaptation	O/Threshold variability
2	Isolated spikes	B/Phasic spiking	A/Tonic spiking
		J/Subthreshold oscillations	H/Class 2
		K/Resonator	
		M/Rebound spiking	
		S/Inhibition-induced spiking	
3	Short bursts	C/Tonic bursting	C/Tonic bursting
			D/Phasic bursting
			E/Mixed mode
			F/Adaptation*
			G/Class 1*
4	Long bursts	D/Phasic bursting	M/Rebound spiking
		N/Rebound bursting	N/Rebound bursting
			S/Inhibition-induced spiking*
			T/Inhibition-induced bursting
5	Long bursts	Q/Depolarizing after-potential	J/Subthreshold oscillations
			K/Resonator*
6	Regular isolated spikes	H/Class 2	P/Bistability
		P/Bistability	Q/Depolarizing after-potential*

Group numbers are arbitrary and do not correspond to cluster numbers in Fig. 9. Models with a different firing pattern in Figs. 4 and 5 than their group are marked with an asterisk. Filter kernel parameters for the groups are shown in Table 8

**Table 8:** Median values of parameters for filter kernels fitted to the six groups described in Table 7 for Izhikevich and AMAT models

Group	*γ*_1_	*γ*_2_	*f*_c,1_[Hz]	*f*_c,2_[Hz]	Δ[ms]
Izhikevich					
1	− 11.98	− 1.41	8.57	61.67	1.35
2	− 1231.41	− 1.01	17.48	19.51	3.60
3	− 154.58	− 1.21	10.51	23.28	0.91
4	− 2273.17	− 1.02	6.70	7.33	6.54
5	54.98	0.19	17.26	403.19	0.74
6	− 1462.74	− 1.02	36.15	38.72	4.24
AMAT					
1	− 90.59	− 1.52	16.03	36.65	0.21
2	2.46	0.50	34.47	161.85	0.22
3	− 34.88	− 1.65	1.53	21.41	0.35
4	− 1436.78	− 1.00	16.06	18.20	1.90
5	32.98	0.29	8.56	407.10	0.27
6	3.89	0.90	12.77	190.14	0.20

Comparing the grouping of models to the spike responses shown in Figs. 4 and 5, we can roughly identify the groups found by *k*-means clustering of filter parameters to firing patterns, as indicated in the right column of Table 7. This classification is far from perfect, as several models show firing patterns different from the groups into which they have been placed, especially for the AMAT class. It should also be noted that the firing patterns are for a single stimulus configuration only and that models may behave differently under other conditions; the *k*-means clustering, on the other hand, is based on a wide range of stimulus conditions.

### Performance of rate models

We evaluate the perfomance of the linear-nonlinear firing rate models by testing them against the corresponding spiking model as described in Section 2.5, using the fit quality *E*_*r*_ as criterium, with *E*_*r*_ = 1 indicating a perfect fit.

The third row of Fig. 6 shows the response to a Poisson spike train with a step in rate from 100s^− 1^ to 300s^− 1^. We use the filters fitted for the same noise regime and synaptic weight and *a*_0_ = 200s^− 1^ and *a*_1_ = 100s^− 1^, corresponding to the step height. For the Tonic spiking case, the firing rate models capture the spiking neuron response very well, with *E*_*r*_ > 0.9 in all cases (see legend). For the Phasic bursting models, we find that the rate models overshoot massively for the Izhikevich variant with no or balanced noise, while the rate models “undershoot” somewhat for the AMAT variant. The stationary rate attained after the step is captured well in all cases. These examples also provide an illustration of how to interpret the fit quality measure *E*_*r*_.

**Fig. 10:**
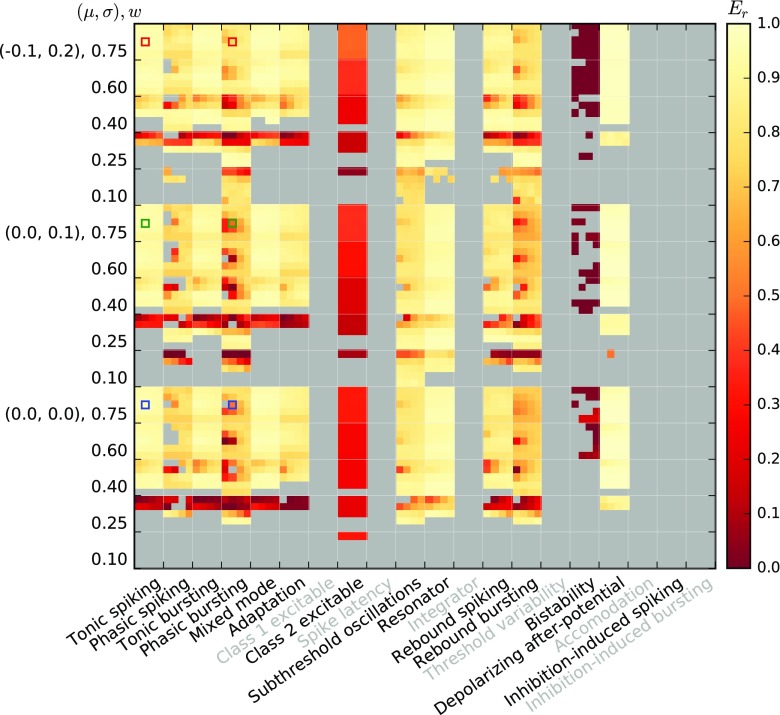
Fit quality *E*_*r*_ for Izhikevich model class responses to a Poisson process with firing rate changing in steps, cf. Table 5, where *E*_*r*_ = 1 indicates a perfect fit. Each major column corresponds to one model, each major row to one of 15 input conditions; thus, each major cell corresponds to one firing-rate estimate *r*_spike_(*t*). Each entry inside a major cell represents the fit against one of 20 filter models obtained for the same *μ*,*σ*,*w* and different combinations of *a*_0_, *a*_1_ as in Fig. 7. Missing results (grey) are either due to models excluded (grey labels) or insufficient responses

**Fig. 11:**
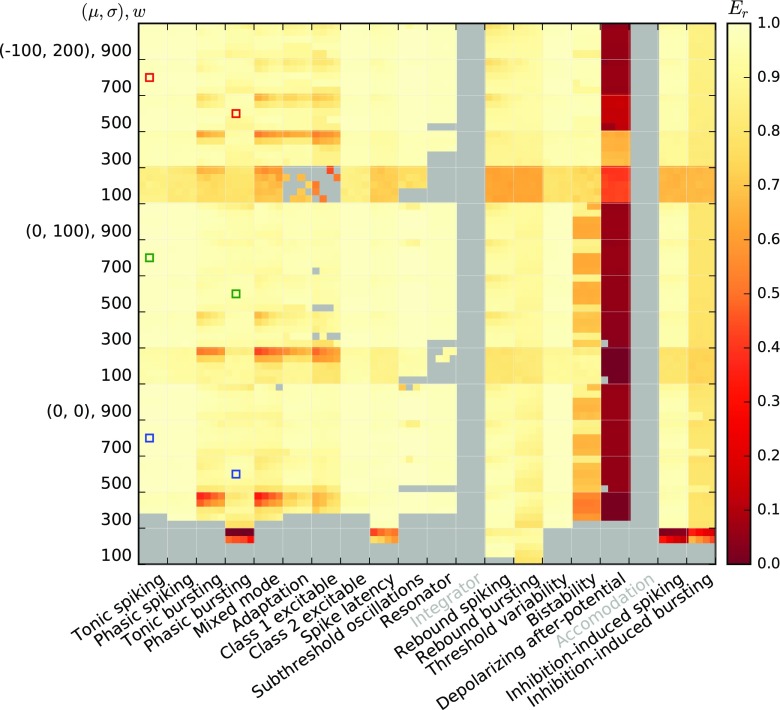
Fit quality *E*_*r*_ for AMAT model class responses to a Poisson process with firing rate changing in steps; all else as in Fig. 10

Figures 10 and 11 show the fit quality observed for responses to Poisson input with piecewise constant rates as described in Section 2.5.1. For each model, we simulate responses under 15 input conditions (three noise regimes (*μ*,*σ*) and five different synaptic weights *w*), yielding 15 firing-rate estimates *r*_spike_(*t*). We then test each firing-rate estimate against the 20 linear-nonlinear rate models obtained for the same *μ*,*σ*,*w* and all *a*_0_,*a*_1_ combinations, yielding 20 fit quality values *E*_*r*_.

For the Izhikevich class, the Inhibition-induced spiking and bursting models, as well as most models with the lowest weight, *w* = 0.1, produce too few spikes to confidently estimate firing rates from the spiking model. We also observe very poor responses for the Bistability model. Class 2 excitable stands out with poor scores, *E*_*r*_ < 0.5, while the remaining models provide reasonable fits, *E*_*r*_ > 0.7 at least for most cases with sufficiently strong weights (*w* ≥ 0.4).

The AMAT model class performs significantly better: Results are available for almost all stimulus conditions except for *w* = 100pA in the absence of noise and all models except the Depolarizing after-potential model yield excellent fits (*E*_*r*_ > 0.9) for almost all conditions.

**Fig. 12:**
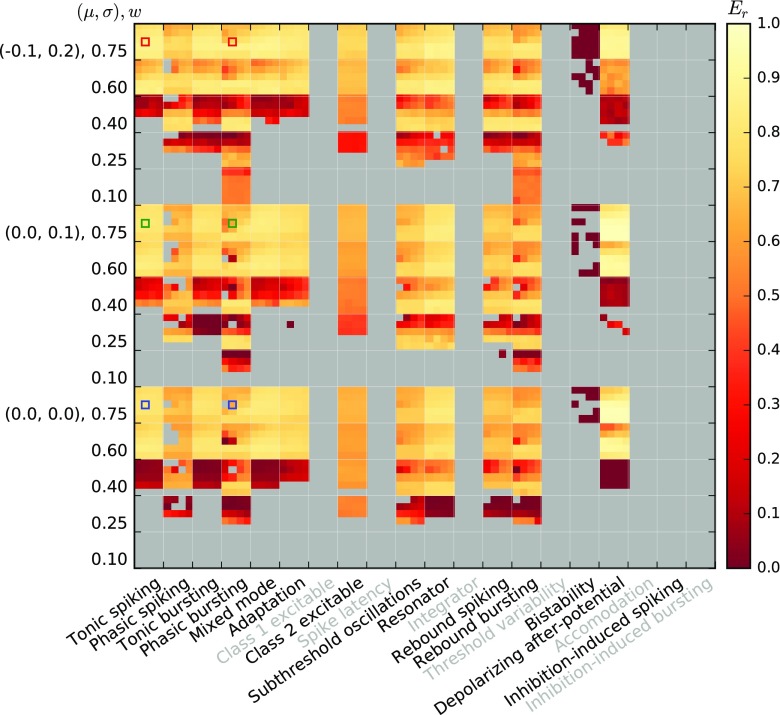
Fit quality *E*_*r*_ for Izhikevich model class responses to the real spike trains from Fig. 3; all else is as in Fig. 10

**Fig. 13:**
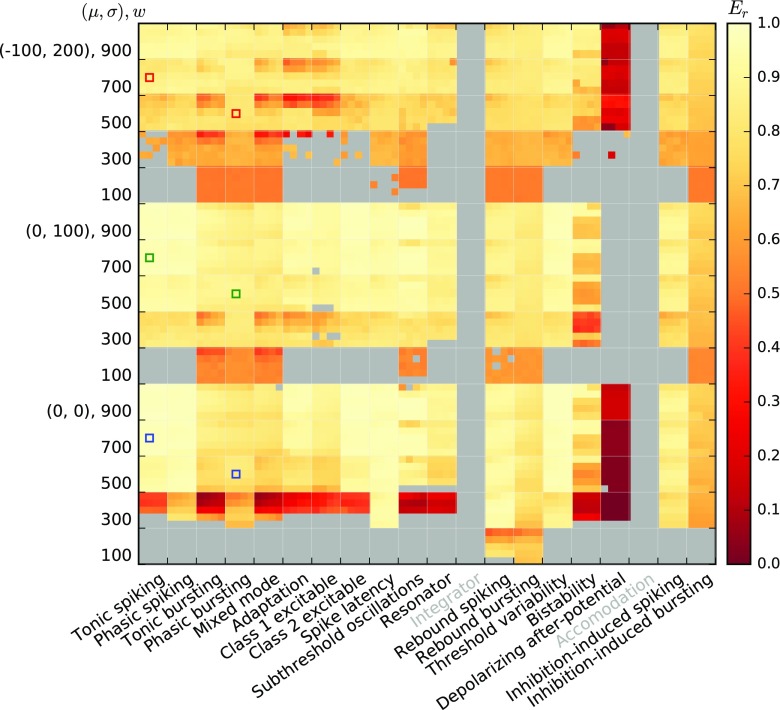
Fit quality *E*_*r*_ for AMAT model class responses to the real spike trains from Fig. 3; all else as in Fig. 10

Figures 12 and 13 show the fit quality *E*_*r*_ for responses to real spike trains from cat retinal ganglion cells as described in Section 2.5.2. We again stimulate under 15 different conditions and obtain the fit quality for each of 20 different linear-nonlinear model fits.

For Izhikevich-class models we find noticeably worse fit quality, mostly *E*_*r*_ < 0.7, with the worst results mostly for the same conditions that also yielded low fit quality in response to Poisson input with piecewise constant rate. The main differences are that the Depolarizing after-potential model, which fitted stepped Poissonian input very well does not perform better than other models for the real spike trains, and that we obtain quality of fit values, albeit very poor ones, for the Inihibition-induced bursting model.

AMAT class responses to real spike trains show all over better fit quality than the Izhikevich class, but also for the AMAT class fit quality is lower in response to real spike trains than to stepped Poisson input. The distribution of good and bad fits is similar to the one observed for stepped Poisson input, with the worst performance for the Depolarizing after-potential model. Furthermore, more models require *w* ≥ 300pA to yield a fit quality result for real spike trains.

**Fig. 14:**
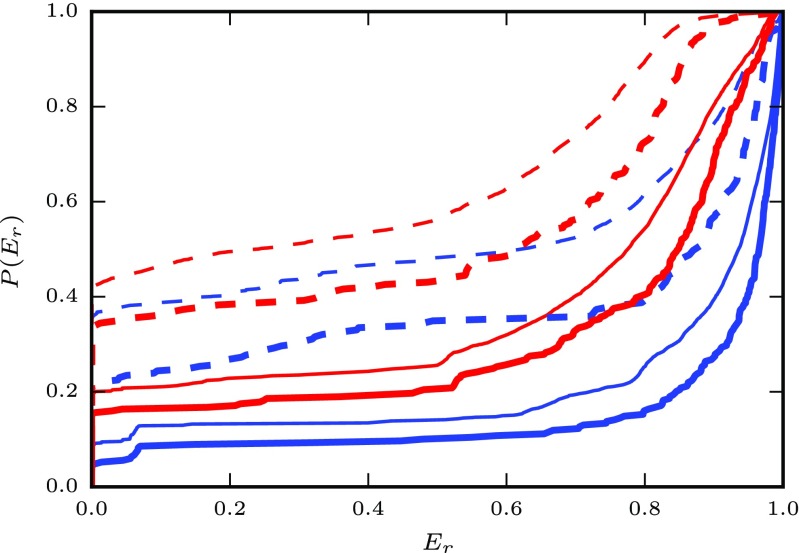
Cumulative distribution *P*(*E*_*r*_) of the fit quality in response to stepped Poisson (blue) and real spike train (red) input for the Izhikevich (dashed) and AMAT classes (solid). Thin lines show the distribution of all individual results from Figs. 10–13, while thick lines show the distribution of optimal scores $E_{r}^{\text {opt}}$ for each input configuration *μ*,*σ*,*w*. If all fits were perfect, *P*(*E*_*r*_) would hug the x axis until jumping to 1 for *E*_*r*_ = 1

We summarize these observations in Fig. 14, which shows the cumulative distribution *P*(*E*_*r*_) of all individual results from Figs. 10–13 (thin lines). This clearly shows that our linear-nonlinear rate models are much more faithful for the AMAT class than for the Izhikevich class, and that within each class, responses to stepped Poisson input are rendered more faithfully than to real spike trains.

These observations pertain to all (up to 20) linear-nonlinear models obtained for each input configuration *μ*,*σ*,*w*. In practice, we would only be interested in the optimal linear-nonlinear model for each input configuration, i.e., for given *μ*,*σ*,*w* we would choose the model with

34 $$ E_{r}^{\text{opt}} = \max_{a_{0}, a_{1}} E_{r} ,  $$

the highest *E*_*r*_ across all *a*_0_,*a*_1_ combinations. The cumulative distribution of fit quality for these optimal models is shown as thick lines in Fig. 14 and provides noticeably better fit quality for stepped Poisson and real train responses for both model classes. Table 9 shows the proportion of cases for which we reach high fit quality ($E_{r}^{\text {opt}}\geq 0.8$) for the optimal models. We find that the linear-nonlinear rate models perform well for the majority of conditions for the AMAT model class, but mostly poorly for the Izhikevich model class.

**Table 9:** Proportion of linear-nonlinear rate models achieving $E_{r}^{\text {opt}}\geq 0.8$ across all model variants, noise regimes and synaptic weights

	Izhikevich	AMAT
Stepped Poisson trains	60%	84%
Real spike trains	28%	59%

### Model generalizations

As we have shown above, for the AMAT model class our linear-nonlinear rate models can capture the responses of spiking neuron models to real spike trains quite accurately. For the Izhikevich class, on the other hand, fits were poorer. Unfortunately, to find the optimal linear-nonlinear model for each input configuration *μ*,*σ*,*w*, we had to test a set of 20 different linear-nonlinear models to then pick the best one. This is impractical. We will now consider how to generalize our linear-nonlinear rate models, so that we can select an optimal model *a priori*.

We consider four different types of generalization:

per model (M): one linear-nonlinear model for each of the 14 Izhikevich class and 18 AMAT class models;
per model and noise (MN): one linear-nonlinear model for each Izhikevich/AMAT model and each noise regime;
per model, noise, and weight (MNW): one linear-nonlinear model for each Izhikevich/AMAT model, each noise regime, and each synaptic weight selected a priori;
MNW selected by stepped response (MNWS): one linear-non-linear model for each Izhikevich/AMAT model, each noise regime, and each synaptic weight selected based on the stepped Poisson test.

For the M and MN generalizations, we exploit that the activation functions *g*(*a*) for many models and conditions scale roughly linear in the synaptic weight. We thus pool the scaled activation function data *g*(*a*)/*w* for a given model across all input conditions (M) or just all synaptic weights for given noise (MN) and fit a single spline $\bar {g}(a)$ to the pooled data. We then use $w\bar {g}(a)$ as activation function in the linear-nonlinear model. For MNW and MNWS we use the original splines fitted directly against measurements.

To generalize the linear kernels, we take the median value for each of the kernel fit parameters *f*_*c*,1_,*f*_*c*,2_,*γ*_1_,*γ*_2_,*d* and use these median parameters as parameters of our generalized kernel; using the median instead of the mean avoids problems with outliers. For M generalization, we take the median across all *μ*,*σ*,*w*,*a*_0_,*a*_1_ combinations, for MN across all *w*,*a*_0_,*a*_1_ for given *μ*,*σ* and for MNW across all *a*_0_,*a*_1_ for given *μ*,*σ*,*w*.

For MNWS generalization, we proceed differently: For each combination of *μ*,*σ*,*w* we select the filter parameters *f*_*c*,1_,*f*_*c*,2_,*γ*_1_,*γ*_2_,Δ which yielded the highest fit quality $E_{r}=E_{r}^{\text {opt}}$ in response to the stepped Poisson input, our test stimulus.

**Fig. 15:**
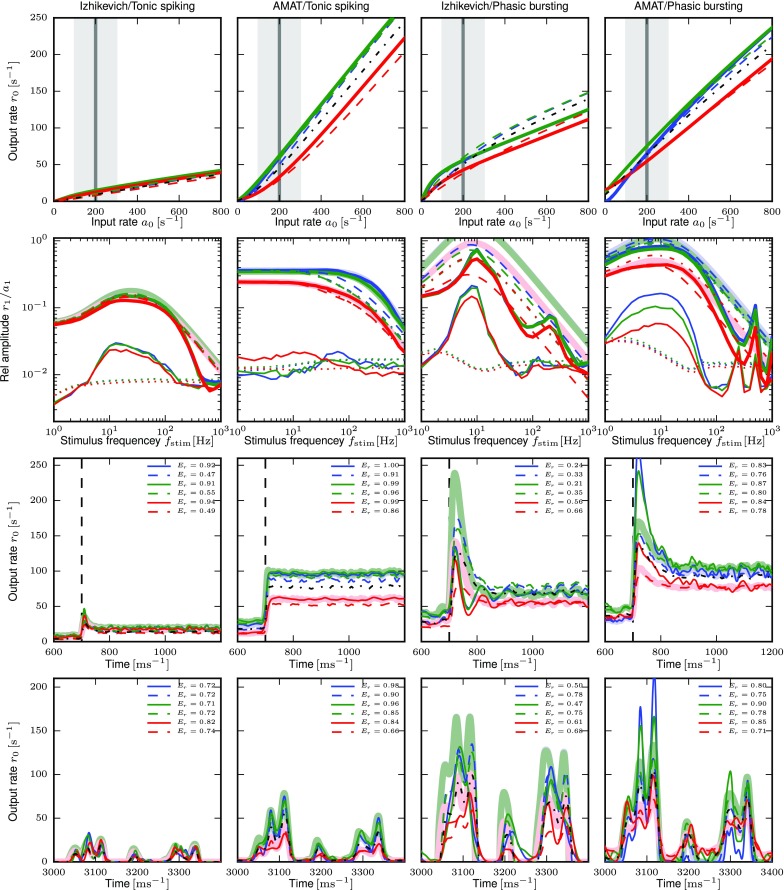
Response properties for exemplary model neurons including responses of model generalizations. This figure is identical to Fig. 6, except that it also shows the response of model generalizations. Top row: activation function for no noise (blue), balanced noise (green) and biased noise (red). Solid lines show original spline fit (also used for MNW and MNWS generalizations), dashed lines responses from MN-generalization and the black dash-dotted line the response for the M-generalization. Second row: Frequency response to sinusoidally modulated Poisson input. Thick solid lines: first harmonic *r*_1_, thin solid lines: second harmonic *r*_2_, dotted lines: significance level *r*_crit_, light thick lines: fitted filter function $\tilde {H}$, dashed lines: filter function for MN-generalizations, dash-dotted lines: filter function for M-generalization. Third row: Response of spiking model (thin solid lines) and rate-model prediction (light thick lines) to Poisson spike trains with rate step. Dashed lines show the response of the MN-generalization, the black dash-dotted line of the M-generalization. Bottom row: Response to real spike trains, 400 ms section starting at 3000 ms with the same line types as for step responses. Connection weight *w* from left to right: 0.75, 700, 0.75, 500

While these generalizations, especially of the M and MN type, may seem rather crude, they perform reasonably, as indicated by the examples shown in Fig. 15. In one case shown there, the Phasic Bursting variant of the Izhikevich model, the model generalizations actually perform better than the specific model fits: The model responses to a firing rate step and to real spikes trains show significant overshoots in low noise regimes (blue and green curves) for the input parameters chosen for illustration (*w* = 0.75, *a*_0_ = 200s^− 1^, *a*_1_ = 100s^− 1^). This is consistent with the overly large amplitudes of the fitted filters. The corresponding generalized model filters fit the experimental data better, avoid the overshoot and thus track the response of the spiking model better.

To systematically quantify the quality of our generalizations, we compute the fit quality in response to stepped Poisson and real spike train input for all input configurations *μ*,*σ*,*w* for each generalization variant. Then

35 $$ \rho_{X} = \left. E_{r}^{\text{X}} \right/ E_{r}^{\text{opt}} $$

measures how close the model generalization *X* is to the optimal linear-nonlinear model, where *ρ*_*X*_ = 1 is best.

**Fig. 16:**
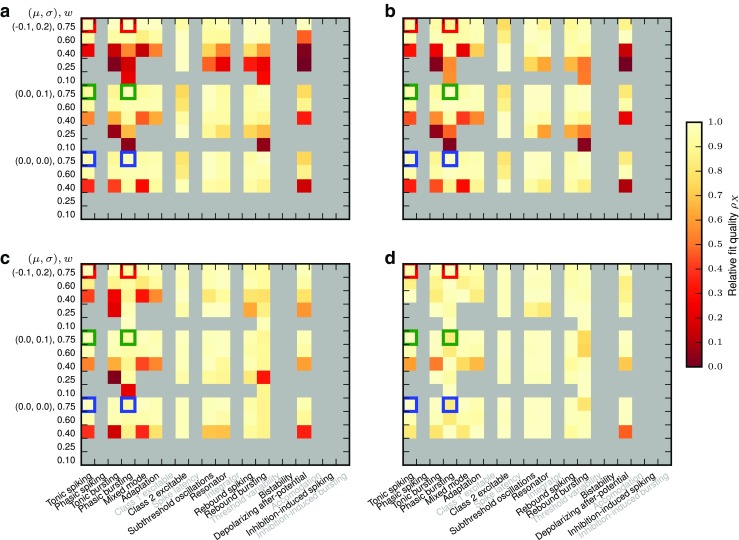
Fit quality relative to optimum, *ρ*_*X*_ for Izhikevich class model generalizations: **a** M, **b** MN, **c** MNW, and **d** MNWS. For details, see text

**Fig. 17:**
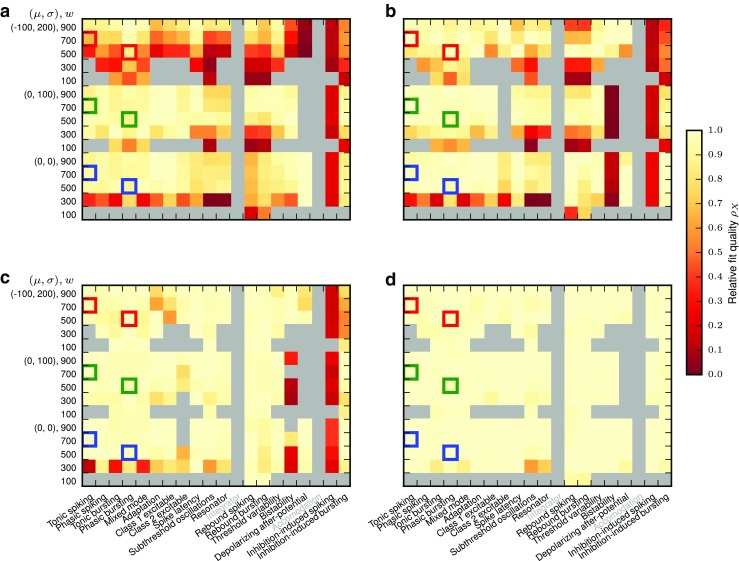
Fit quality relative to optimum, *ρ*_*X*_ for AMAT class model generalizations: **a** M, **b** MN, **c** MNW, and **d** MNWS. For details, see text

Results are shown in Figs. 16 and 17. The coarser the generalization, the more frequently do we observe low generalization quality *ρ*_*X*_. Interestingly, differences between the various generalizations are larger for the AMAT class than for the Izhikevich class, and generalization seems to fail for the AMAT class mostly for biased noise and Inhibition-induced spiking. For the Izhikevich class, on the other hand, generalization mostly fails for low synaptic weights.

The most important observation, though, is that MNWS generaliztion works well, with *ρ*_MNWS_ > 0.9 in almost all cases for both model classes. This means that by selecting a filter model based on a fixed stepped Poisson protocol, we will obtain a linear-nonlinear rate model that is close to the optimal model for given noise regime and synaptic weight when applied to real neuronal dynamics.

Combined with the observation from Table 9 that the optimal model will provide a good approximation to actual neuronal firing rates in roughly two thirds of all conditions, we can thus use our fitting approach together with the stepped Poisson test to select a reasonably reliable linear-nonlinear neuron model.

## Discussion

In this paper we numerically investigated the response properties of two neuron model classes, the Izhikevich model and the AMAT model, to noisy spiking input. Both neuron models can reproduce a wide range of experimentally observed spike response patterns when stimulated with current injections. However, how these neurons behave with more natural synaptic inputs has so far not been studied systematically. We considered three different background noise regimes, one with no background noise at all, one balanced and one biased with enough background noise to put neurons in a spontaneously active state at low output rates. The first scenario can be considered to represent the situation in slice preparations, the other two correspond to neurons embedded in a network with ongoing excitatory and inhibitory activity. The stimulus spikes were modeled as stationary and sinusoidally modulated excitatory Poisson input spike trains, mimicking afferent inputs from sensory pathways with different synaptic connection strengths *w*.

### Responses to spike input

We found that the response complexity observed under current injection collapses to only a few response types when the neurons are driven by stationary or sinusiodally modulated Poisson input. This is not entirely surprising, since some of the models are parametrically quite similar, and variations in response behavior to current stimulation depend on very specific current injection patterns that are not realizable in terms of Poisson spike inputs. Still, actual neurons receive inputs that often are well-described by Poissonian statistics and this can thus be considered the functionally more relevant input scenario. It is hence of interest to see which, possibly quite different, neuron models behave approximately equivalent.

The respective groupings for Izhikevich and AMAT are all-in-all very different. In particular, direct comparison of the individual corresponding neuron models reveals completely different response properties for most neuron models. This is in part explained by the differences in subthreshold dynamics which are linear for the AMAT model but nonlinear for the Izhikevich model. Individual spikes thus have quite different effects in the two models: Any input spike to an AMAT neuron will always evoke the same postsynaptic membrane-potential response and these responses simply superimpose due to the subthreshold linearity. For the Izhikevich models, on the other hand, the postsynaptic response depends intricately on the value of the dynamic variables, such as the membrane potential, and the effect of several incoming excitatory spikes of same weight at one moment might be smaller than that of just one such spike at another moment. These differences hence make it hard, or even impossible, to set up synaptic weights *w* for the two neuron model classes that are directly comparable.

We therefore chose to gauge synaptic strengths in terms of the minimal weight *w*_*𝜃*_ needed to evoke a spike from rest, cf. Section 2.2, and to use weights spanning roughly from 10% to 75% of *w*_*𝜃*_. This allowed us to quantify and compare input coupling strength within and between model classes. In general we observed that output rates for Izhikevich neurons were much lower than for AMAT neurons for the same input frequency and relative synaptic strength. It is therefore possible that model classes might become more similar if the Izhikevich neurons were driven at higher input rates or at other background noise levels, although we did not observe such a trend.

We observed here that neuron models can show very similar responses to spike input, even though they show very different responses to current injections, and in particular that models of different mathematical nature, showing *identical* current responses can respond very differently to spiking input. Given that neurons are mainly driven by spike input *in vivo*, this raises the intriguing question of how valuable a classification of neuronal response types based purely on current injection experiments is. While *in vitro* characterization using carefully crafted current injections is an important tool to classify neuronal cell types, it appears that a systematic classification based on a neuron’s response to spiking input may be required to select suitable neuron models for spiking and rate-based network models.

### Firing-rate models

In the second part of the paper, we made use of the measured stationary and frequency responses to fit linear-nonlinear firing-rate models to the data. It has previously been shown that the firing-rate dynamics in response to complex spiking input can be well described by such models (Paninski et al. 2004; Ostojic and Brunel 2011; Weber and Pillow 2017; Østergaard et al. 2018). In particular, Nordlie et al. (2010) studied simple leaky integrate-and-fire (LIF) models with strong current-based synapses. They showed that a lowpass fit to the frequency response together with the nonlinear activation function yielded linear-nonlinear rate models that predicted responses to arbitrary inputs with high accuracy. Heiberg et al. (2013) adapted this approach and studied two LIF-like models, one with current-based, the other with conductance-based synapses, that were fit to actual data recorded from cat and macaque LGN in response to retinal stimulation. They found the performance of linear-nonlinear rate models to be good as well.

Here, we presented results of the same basic approach for the Izhikevich and AMAT neuron model classes. Frequency responses were in most cases more complex than simple lowpass behavior and we employed fits to bandpass filters that better capture the non-monotonous passband structure observed in simulations. We then used novel test stimuli, i.e., step responses and more structured, highly variable spike input sampled from actual recordings of retinal ganglion cells (Casti et al. 2008) to study rate-model performance. The main finding is that the AMAT neuron model class is approximated much better than the Izhikevich class by our linear-nonlinear rate models: for the former, good rate model responses (*E*_*r*_ ≥ 0.8) were obtained in 64% of all cases tested, while the latter provided such good results in only 15% of cases tested. This difference might again be explained by the fact that the AMAT model class has subthreshold linear dynamics. However, the AMAT class is not completely linear either, because its firing threshold depends on the history of the membrane-potential dynamics. Therefore, neuronal transfer is not expected to be linear in any model class.

Some of the model variants gave consistently poor results, typically those that show very nonlinear behavior in response to direct current stimulation, e.g., the *Bistability*, *Inhibition-induced spiking* and *Inhibition-induced bursting* models for the Izhikevich class, and the *Depolarizing afterpotential* and *Inhibition induced spiking* models for the AMAT class.

To estimate the effects of linearity on rate-model performance, we measured the linearity of the stationary response function *r*_0_(*a*_0_) in terms of *L*_1_, cf. Eq. (12). If the stationary response function is linear, the activation function *g*(⋅) is also linear and only its slope is relevant, independent of the working point, cf. Section 2.4. We computed *L*_1_ for all background noise regimes as a function of synaptic strength *w* and working point *a*_0_, and find that the AMAT model generally is more linear than the Izhikevich model with respect to *L*_1_. We further find that the linearity measure *L*_1_ does not predict rate-model performance (data not shown). Thus, a nonlinear activation function does not imply poor rate-model performance, nor does linearity in terms of *L*_1_ necessarily predict a good rate-model performance.

Furthermore, despite exploration of many potential performance predictors, we were unable to identify any single quantity or group of quantities that reliably predicted whether the response of a neuron model in a given input regime could be captured well by a linear-nonlinear rate model. We found, though, that a relative simple protocol, testing the rate model’s performance in response to a Poisson spike train input with piecewise constant rate (stepped Poisson), allowed us to reliably identify rate models that render spiking neuron model responses to realistic spike input with reasonable accuracy.

### Application to network modeling

We have shown that the firing-rate responses of the widely-used Izhikevich model (Izhikevich 2003b) and in particular of the award-winning augmented multi-timescale adaptive threshold (AMAT) model (Yamauchi et al. 2011; Jolivet et al. 2008) can be captured by linear-nonlinear firing-rate models with bandpass filters fitted to spiking neuron responses through a systematic, automated process. We have further shown that these models can be generalized across a wide range of input conditions without excessive loss of fidelity, and that optimal parameter sets can be chosen using simple test stimuli. Furthermore, since we use a bandpass filter in sum form, it can be represented by a system of two first-order differential equations, which is straightforward to integrate into standard formalisms for rate-based network models (see, e.g., Nordbø et al. 2007).

This suggests the following approach to improve rate-based neuronal network models based on our findings. Assuming a model with two neuronal populations, start by selecting for each population the AMAT (or Izhikevich) model variant that best matches the response of individual neurons of each population to spiking input. Then apply the fitting procedure described in this paper (Section 2.4) to obtain the parameters of the nonlinear activation function and the linear bandpass filter using test stimuli covering the expected dynamic range of your network model. From the large set of fits obtained, either select individual fits based on a simple test protocol (Section 3.4) or generalized at a suitable level (Section 3.5), and apply the model parameters thus obtained to the differential-equation representation of the bandpass filter (Section 2.4.1).

The approach presented here may thus contribute to bringing rate-based network modeling closer to the reality of biological neuronal networks. While a systematic delineation of the range of validity of the linear-nonlinear models described here for network modeling is beyond the scope of this paper, we consider the generalization results in Figs. 16 and 17 an indicator: Linear-nonlinear models will not be useful in cases where responses are insufficient (gray areas in Figs. 10–13 and 16–17); we also observed poorer performance for spiking patterns that deviate strongly from tonic behavior (e.g., phasic bursting, bistability, depolarizing afterpotential). But good generalization scores, in particular where coinciding with good scores for individual conditions (Figs. 10–13) suggest that in these cases our linear-nonlinear model provides a faithful representation for the rate dynamics of the underlying spike responses, and hence a strong potential for good performance also on the network level.

## Appendix

The AMAT model as specified by Equations 1–3, 16, 17, and A1–A7 of Yamauchi et al. (2011) models input as instantaneous jumps in the membrane potential (*δ*-synapses) or as *α*-function post-synaptic currents (PSCs). We adapt the model to synapses injecting exponentially decaying PSCs and add a piecewise-constant input current. We further re-parameterize the model from membrane resistance *R* and time constant *τ*_*m*_ to membrance capacitance *C* and time constant *τ*_*m*_ by setting *R* = *τ*_*m*_/*C*; this brings the model equations in line with conventions for the NEST Simulator (Gewaltig and Diesmann 2007; Morrison et al. 2007). The resulting set of differential equations are then integrated using the exact integration technique (Rotter and Diesmann 1999; Plesser and Diesmann 2009).

The AMAT model with two adaptive thresholds (AMAT2 model), two exponential-current synapses and piecewise-constant input current is defined by the following equations

36 $$\begin{array}{@{}rcl@{}} \dot{V} &=& -\frac{V}{\tau_{m}} + \frac{I}{C} \end{array} $$

37 $$\begin{array}{@{}rcl@{}} \dot{\theta}_{1} &=& -\frac{\theta_{1}}{\tau_{1}} + \alpha_{1}\sum\limits_{k}\delta(t-\hat{t}_{k}) \end{array} $$

38 $$\begin{array}{@{}rcl@{}} \dot{\theta}_{2} &=& -\frac{\theta_{2}}{\tau_{2}} + \alpha_{2}\sum\limits_{k}\delta(t-\hat{t}_{k}) \end{array} $$

39 $$\begin{array}{@{}rcl@{}} \ddot{\theta}_{V} + \frac{2\dot{\theta}_{V}}{\tau_{V}} + \frac{\theta_{V}}{{\tau_{V}^{2}}} &=& \beta\dot{V} = -\frac{\beta V}{\tau_{m}} + \frac{\beta I}{C} . \end{array} $$

Here, $\hat {t}_{k}$ are the times of the spikes fired by the neuron itself. A spike is fired whenever

40 $$ V(t)\geq\theta(t) = \omega + \theta_{1} + \theta_{2} + \theta_{V} $$

provided the neuron is not refractory. To be precise, let $\hat {t}_{k}$ be the time of the most recent spike and *τ*_ref_ the duration of the absolute refractory period. Then the time of the next spike is given by

41 $$ \hat{t }_{k + 1} = \min \{t>\hat{t}_{k}+\tau_{{\text{ref}}}|V(t)\geq\theta(t)\} . $$

Note that the membrane potential is *not* reset upon a spike.

To obtain first-order differential equations for *𝜃*_*V*_ (Rotter and Diesmann 1999), we define

42 $$ \eta = \dot{\theta}_{V}+\frac{\theta_{V}}{\tau_{v}} $$

and arrive at the equations

43 $$\begin{array}{@{}rcl@{}} \dot{\eta} &=& -\frac{\beta V}{\tau_{m}} + \frac{\beta I}{C} \end{array} $$

44 $$\begin{array}{@{}rcl@{}} \dot{\theta}_{V} &=& \eta - \frac{\theta_{V}}{\tau_{V}} \end{array} $$

as can be verified by inserting the first differential equation into the time derivative of the second and comparing with Eq. (39).

We treat spike input as follows: We assume that the PSC evoked by input through any synapse on the neuron will decay either with time constant *τ*_*E*_ or *τ*_*I*_. Let $\{(\hat {t}_{X, j}, w_{X,j})\}$ be the set of all spike arrival times and weights of spikes arriving through synapses with time constant *τ*_*X*_ where *X* ∈{*E*,*I*}. If we further assume that all PSCs are independent of each other, we can express the total input as

45 $$ I(t) = I_{E}(t) + I_{I}(t) + {{I}_{\text{{ext}}}}(t) $$

where

46 $$ I_{X}(t) = \sum\limits_{j} w_{X,j} e^{-\frac{t-\hat{t}_{X,j}}{\tau_{X}}} {\Theta}(t-\hat{t}_{X,j}) $$

and *I*_ext_(*t*) is a piecewise-constant external current and Θ(*t*) the Heaviside step function. We will commonly refer to *I*_*E*_ as excitatory and *I*_*I*_ as inhibitory input. For efficient integration of the synaptic currents (Plesser and Diesmann 2009), we describe them by differential equations

47 $$ \dot{I}_{X}+\tau_{X} I_{X} = \sum\limits_{j} w_{X,j} \delta(t-\hat{t}_{X,j}) . $$

We can summarize the resulting system of eight first-order linear differential equations as

48 $$ \dot{\mathbf{y}} = \mathbf{A}\mathbf{y} + \mathbf{x} $$

with state and input vectors

49 $$ \mathbf{y} = \left( \begin{array}{ccccccccc} I_{\text{ext}}\\ I_{E} \\ I_{I} \\ V \\ \theta_{1} \\ \theta_{2} \\ \dot{\theta}_{V}+\frac{\theta_{V}}{\tau_{V}}\\ \theta_{V} \end{array}\right) \qquad \mathbf{x} = \left( \begin{array}{ccccccccc} {\Delta}{{I}_{\text{{ext}}}}(t)\\ {\sum}_{j} {w}_{j}^{E} \delta(t-\hat{t}_{j}^{E}) \\ {\sum}_{j} {w}_{j}^{I} \delta(t-\hat{t}_{j}^{I}) \\ 0 \\ \alpha_{1} {\sum}_{k} \delta(t-\hat{t}_{k}) \\ \alpha_{2} {\sum}_{k} \delta(t-\hat{t}_{k}) \\ 0 \\ 0 \end{array}\right) $$

and system matrix

50 $$ \mathbf{A} =\left( \begin{array}{cccccccccc} 0 & 0 & 0 & 0 & 0 & 0 & 0 & 0 \\ 0 & -\frac{1}{\tau_{E}} & 0 & 0 & 0 & 0 & 0 & 0 \\ 0 & 0 & -\frac{1}{\tau_{I}} & 0 & 0 & 0 & 0 & 0 \\ 0 & \frac{1}{C} & \frac{1}{C} & -\frac{1}{\tau_{m}} & 0 & 0 & 0 & 0 \\ 0 & 0 & 0 & 0 & -\frac{1}{\tau_{1}} & 0 & 0 & 0 \\ 0 & 0 & 0 & 0 & 0 & -\frac{1}{\tau_{2}} & 0 & 0 \\ 0 & \frac{\beta}{C} & \frac{\beta}{C} & -\frac{\beta}{\tau_{m}} & 0 & 0 & -\frac{1}{\tau_{V}} & 0 \\ 0 & 0 & 0 & 0 & 0 & 0 & 1 & -\frac{1}{\tau_{V}} \end{array}\right) . $$

Δ*I*_ext_(*t*) represents the jumps in the piecewise constant external input current.

We can solve this system exactly (to the limits of machine precision) on a fixed time grid *t*_*j*_ = *j**h* for *h* > 0 using exact integration (Rotter and Diesmann 1999), provided that *I*_ext_(*t*) only changes at grid points *t*_*j*_, i.e., ${\Delta }{{I}_{\text {{ext}}}}(t)={\sum }_{j} \delta I_{j} \delta (t-t_{j})$. Starting from the initial state *y*_0_ = **y****(****t** **=** **0****)**, exact integration updates the state according to

51 $$ \mathbf{y_{j + 1}}= \mathbf{A}\mathbf{y_{j}} + \mathbf{x_{j + 1}} $$

where **A** is the propagator matrix

52 $$\mathbf{P} = e^{\mathbf{A}h} = \sum\limits_{j = 0}^{\infty} \frac{(\mathbf{A}h)^{j}}{j!} . $$

The $\delta (t_{j}-\hat {t}_{k})$-functions in the input vector *x*(*t*) are replaced by Kronecker symbol *δ*_*j*,*k*_ upon discretization, restricting spike times to the time grid; see Morrison et al. (2007) for an extension to off-grid spikes.

The propagator matrix **P** can be obtained numerically, e.g., using the expm functions provided by SciPy or Matlab, using an algorithm due to Higham (2005). These methods can, though, fail under certain circumstances (Moler 2012; Al-Mohy and Higham 2009) and we have not performed any systematic tests regarding their reliability with respect to model neuron dynamics. We used the Mathematica symbolic algebra system (Wolfram 1999) to obtain an explicit expression for the propagator matrix **P** and generate C++-code for the matrix elements. Note that the expression obtained in this way requires that all time constants except *τ*_1_ and *τ*_2_ differ, i.e., *τ*_*m*_≠*τ*_*V*_≠*τ*_*E*_≠*τ*_*I*_. Other expressions for **P** pertain if any two time constants are equal. The resulting model is implemented in NEST as amat2_psc_exp.

While our implementation of the AMAT2 model is based on the equations given by Yamauchi et al. (2011), we found that we needed to modify some model parameters to reproduce the responses in Figs. 6 and 7 of that paper:

- all models: membrane time constant *τ*_*m*_ = 10 ms instead of *τ*_*m*_ = 5 ms
- *subthreshold oscillations*: *α*_1_ = 1, *β* = 0.2 instead of *α*_1_ = 10, *β* = 0.1
- *resonator*: *β* = 0.5 instead of *β* = 0.1
- *threshold variability*: *β* = − 0.5 instead of *β* = − 0.1

Parameters used in our model are given in Table 4.

We further needed to make some adjustments to stimulus parameters to reproduce Figs. 6 and 7 in Yamauchi et al. (2011):

- *tonic spiking*: *I*_*c*_ = 0.118 nA instead of *I*_*c*_ = 0.15 nA
- *phasic spiking*: *I*_*c*_ = 0.1 nA instead of *I*_*c*_ = 0.08 nA
- *phasic bursting*: *I*_*c*_ = 0.1 nA instead of *I*_*c*_ = 0.08 nA
- *class 1 excitable*: *d**I*/*d**t* = 150 pA/s instead of *d**I*/*d**t* = 2.5 pA/s
- *class 2 excitable*: *d**I*/*d**t* = 150 pA/s instead of *d**I*/*d**t* = 2.5 pA/s
- *latency*: *I*_*p*_ = 1 nA instead of *I*_*c*_ = 0.58 nA
- *subthreshold oscillations*: *I*_*p*_ = 0.8 nA instead of *I*_*p*_ = 0.2 nA
- *resonator*: *I*_*p*_ = 0.6 nA instead of *I*_*p*_ = 0.36 nA
- *integrator*: *I*_*p*_ = 0.45 nA instead of *I*_*p*_ = 0.28 nA
- *rebound spiking*: *I*_*p*_ = − 1.5 nA instead of *I*_*p*_ = − 0.6 nA
- *rebound bursting*: *I*_*p*_ = − 1.5 nA instead of *I*_*p*_ = − 0.6 nA
- *threshold variability*: *I*_*p*_ = ± 0.35 nA instead of *I*_*p*_ = ± 0.2 nA
- *bistability*: *I**S**I* = 222 ms instead of *I**S**I* = 22 ms
- *depolarizing after-potential*: *I*_*p*_ = 0.5 nA instead of *I*_*p*_ = 0.2 nA
- *accomodation*: *d**I*/*d**t* = 0.54 nA/s, 2.43 nA/s, Δ_ramp_ = 180 ms, 40 ms instead of *d**I*/*d**t* = 1 nA/s, 4.5 nA/s, Δ_ramp_ = 90 ms, 20 ms
- *inhibition-induced spiking*: *I*_*p*_ = − 0.4 nA instead of *I*_*p*_ = − 0.3 nA
- *inhibition-induced bursting*: *I*_*p*_ = − 0.3 nA instead of *I*_*p*_ = − 0.16 nA

The changes in stimulation currents are only relevant for the illustrative Fig. 1 and do not directly affect the remainder of the results presented here.

We can only speculate about why these parameters changes were necessary. We inferred the time axis of Figs. 6 and 7 of Yamauchi et al. (2011) from the scale bars given in panel A of each figure. This clearly indicates that pulse/ramp durations given in Table 1 of that paper are inconsistent with the figures for, e.g., *bistability* (Fig. 6F) and *accomodation* (Fig. 7I). Concerning the membrane time constant *τ*_*m*_, the authors tested various values of this parameter (Yamauchi et al. 2011, p. 2, right column) and this may have led to a mix-up.

## Footnotes

1 Strictly speaking, this is a *functional*, not a function, but we ignore such mathematical detail here as we focus on instantaneous transformations in what follows.

2 The current generated is stepwise constant during each *d**t* = 0.1ms time step, with Gaussian-distributed amplitude.

3 We also explored combining the terms in product form

$$\tilde{H}_{0,\text{Prod}}(f) = \gamma_{1} e^{-2\pi if{\Delta}} \frac{1}{1+ i\frac{f}{f_{\text{c,1}}}} \left( 1 - \frac{\gamma_{2}}{1+ i\frac{f}{f_{\text{c,2}}}} \right) , $$

but did not observe significantly different results.

4 Each parameter set consists of *f*_*c*,1_, *f*_*c*,2_, *γ*_1_, *γ*_2_, and Δ. To compress widely scattering data, we transformed *f*_*c*,1_, *f*_*c*,2_, and Δ using *α*(*x*) = log 10*x* and *γ*_1_ and *γ*_2_ using *β*(*x*) = sgn*x* log 10100|*x*| before applying *k*-means clustering.

## References

[CR1] Al-Mohy AH, Higham NJ (2009). A new scaling and squaring algorithm for the matrix exponential. SIAM Journal on Matrix Analysis and Applications.

[CR2] Avermann M, Tomm C, Mateo C, Gerstner W, Petersen CCH (2012). Microcircuits of excitatory and inhibitory neurons in layer 2/3 of mouse barrel cortex. Journal of Neurophysiology.

[CR3] Blomquist P, Devor A, Indahl UG, Ulbert I, Einevoll GT, Dale AM (2009). Estimation of thalamocortical and intracortical network models from joint thalamic single-electrode and cortical laminar-electrode recordings in the rat barrel system. PLoS Computational Biology.

[CR4] Brette R, Gerstner W (2005). Adaptive exponential integrate-and-fire model as an effective description of neuronal activity. Journal of Neurophysiology.

[CR5] Brunel N (2000). Dynamics of sparsely connected networks of excitatory and inhibitory spiking neurons. Journal of Computational Neuroscience.

[CR6] Brunel N, Chance FS, Fourcaud N, Abbott LF (2001). Effects of synaptic noise and filtering on the frequency response of spiking neurons. Physical Review Letters.

[CR7] Burkitt A. N. (2006). A Review of the Integrate-and-fire Neuron Model: I. Homogeneous Synaptic Input. Biological Cybernetics.

[CR8] Burkitt A. N. (2006). A review of the integrate-and-fire neuron model: II. Inhomogeneous synaptic input and network properties. Biological Cybernetics.

[CR9] Casti A, Hayot F, Xiao Y, Kaplan E (2008). A simple model of retina-LGN transmission. Journal of Computational Neuroscience.

[CR10] Chance FS, Abbott LF, Reyes AD (2002). Gain modulation from background synaptic input. Neuron.

[CR11] Coombes S (2005). Waves, bumps, and patterns in neural field theories. Biological Cybernetics.

[CR12] Ermentrout B (1998). Neural networks as spatio-temporal pattern-forming systems. Reports on Progress in Physis.

[CR13] FitzHugh R (1961). Impulses and physiological states in theoretical models of nerve membrane. Biophysical Journal.

[CR14] Funke K, Wörgötter F (1997). On the significance of temporally structured activity in the dorsal lateral geniculate nucleus (LGN). Progress in Neurobiology.

[CR15] Gerstein GL, Mandelbrot B (1964). Random walk models for the spike activity of a single neuron. Biophysical Journal.

[CR16] Gewaltig MO, Diesmann M (2007). NEST (NEural Simulation Tool). Scholarpedia.

[CR17] Haider B, Häusser M, Carandini M (2013). Inhibition dominates sensory responses in the awake cortex. Nature.

[CR18] Heiberg T, Kriener B, Tetzlaff T, Casti A, Einevoll GT, Plesser HE (2013). Firing-rate models capture essential response dynamics of LGN relay cells. Journal of Computational Neuroscience.

[CR19] Helias M, Kunkel S, Masumoto G, Igarashi J, Eppler JM, Ishii S, Fukai T, Morrison A, Diesmann M (2012). Supercomputers ready for use as discovery machines for neuroscience. Frontiers in Neuroinformatics.

[CR20] Higham NJ (2005). The scaling and squaring method for the matrix exponential revisited. SIAM Journal on Matrix Analysis and Applications.

[CR21] Hodgkin AL, Huxley AF (1952). A quantitative description of membrane current and its application to conduction and excitation in nerve. Journal of Physiology.

[CR22] Ikegaya Y, Sasaki T, Ishikawa D, Honma N, Tao K, Takahashi N, Minamisawa G, Ujita S, Matsuki N (2013). Interpyramid spike transmission stabilizes the sparseness of recurrent network activity. Cerebral Cortex.

[CR23] Izhikevich, E.M. (2003a). Figure 1.m MATLAB script. http://www.izhikevich.org/publications/figure1.m, last accessed 18 Aug 2017.

[CR24] Izhikevich E.M. (2003). Simple model of spiking neurons. IEEE Transactions on Neural Networks.

[CR25] Izhikevich EM (2004). Which model to use for cortical spiking neurons?. IEEE Transactions on Neural Networks.

[CR26] Izhikevich EM (2010). Hybrid spiking models. Philosophical Transactions. Series A, Mathematical, Physical, and Engineering Sciences.

[CR27] Jansen BH, Rit VG (1995). Electroencephalogram and visual evoked potential generation in a mathematical model of coupled cortical columns. Biological Cybernetics.

[CR28] Johannesma P. I. M. (1968). Diffusion Models for the Stochastic Activity of Neurons. Neural Networks.

[CR29] Jolivet R, Rauch A, Lüscher HR, Gerstner W (2006). Predicting spike timing of neocortical pyramidal neurons by simple threshold models. Journal of Computational Neuroscience.

[CR30] Jolivet R, Schürmann F, Berger TK, Naud R, Gerstner W, Roth A (2008). The quantitative single-neuron modeling competition. Biological Cybernetics.

[CR31] Jones, E., Oliphant, T., Peterson, P., et al. (2001). SciPy: open source scientific tools for Python. http://www.scipy.org/, [Online; Accessed 09 March 2015].

[CR32] Kobayashi R, Tsubo Y, Shinomoto S (2009). Made-to-order spiking neuron model equipped with a multi-timescale adaptive threshold. Frontiers in Computational Neuroscience.

[CR33] Kunkel S, Schmidt M, Eppler JM, Plesser HE, Masumoto G, Igarashi J, Ishii S, Fukai T, Morrison A, Diesmann M, Helias M (2014). Spiking network simulation code for petascale computers. Frontiers in Neuroinformatics.

[CR34] Lapicque L (1907). Considérations préalables sur la nature du phénomene par lequel l’électricité excite les nerfs. Journal de Physiologie et de Pathologie Générale.

[CR35] Lefort S, Tomm C, Sarria JCF, Petersen CCH (2009). The excitatory neuronal network of the C2 barrel column in mouse primary somatosensory cortex. Neuron.

[CR36] Mainen ZF, Sejnowski TJ (1996). Influence of dendritic structure on firing pattern in model neocortical neurons. Nature.

[CR37] Markram H, Toledo-Rodriguez M, Wang Y, Gupta A, Silberberg G, Wu C (2004). Interneurons of the neocortical inhibitory system. Nature Reviews Neuroscience.

[CR38] Moler, C. (2012). A balancing act for the matrix exponential. http://blogs.mathworks.com/cleve/2012/07/23/a-balancing-act-for-the-matrix-exponential/.

[CR39] Morris C, Lecar H (1981). Voltage oscillations in the barnacle giant muscle fiber. Biophysical Journal.

[CR40] Morrison A, Straube S, Plesser HE, Diesmann M (2007). Exact subthreshold integration with continuous spike times in discrete time neural network simulations. Neural Computation.

[CR41] Muller, E., Davison, A.P., Brizzi, T., Bruederle, D., Eppler, J.M., Kremkow, J., Pecevski, D., Perrinet, L., Schmuker, M., Yger, P. (2009). NeuralEnsemble.Org: Unifying neural simulators in Python to ease the model complexity bottleneck. In *Frontiers in Neuroscience Conference Abstract: Neuroinformatics 2009*. 10.3389/conf.neuro.11.2009.08.104.

[CR42] Nordbø Ø, Wyller J, Einevoll GT (2007). Neural network firing-rate models on integral form: effects of temporal coupling kernels on equilibrium-state stability. Biological Cybernetics.

[CR43] Nordlie E, Tetzlaff T, Einevoll GT (2010). Rate dynamics of leaky integrate-and-fire neurons with strong synapses. Frontiers in Computational Neuroscience.

[CR44] Østergaard J, Kramer MA, Eden UT (2018). Capturing spike variability in noisy Izhikevich neurons using point process generalized linear models. Neural Computation.

[CR45] Ostojic S, Brunel N (2011). From spiking neuron models to linear-nonlinear models. PLoS Computational Biology.

[CR46] Paninski L, Pillow JW, Simoncelli EP (2004). Maximum likelihood estimation of a stochastic integrate-and-fire neural encoding model. Neural Computation.

[CR47] Pedregosa F, Varoquaux G, Gramfort A, Michel V, Thirion B, Grisel O, Blondel M, Prettenhofer P, Weiss R, Dubourg V, Vanderplas J, Passos A, Cournapeau D, Brucher M, Perrot M, Duchesnay E (2011). Scikit-learn: machine learning in python. Journal of Machine Learning Research.

[CR48] Petersen C, Crochet S (2013). Synaptic computation and sensory processing in neocortical layer 2/3. Neuron.

[CR49] Pillow JW, Paninski L, Uzzell VJ, Simoncelli EP, Chichilnisky EJ (2005). Prediction and decoding of retinal ganglion cell responses with a probabilistic spiking model. Journal of Neuroscience.

[CR50] Plesser HE, Diesmann M (2009). Simplicity and efficiency of integrate-and-fire neuron models. Neural Computation.

[CR51] Plesser, H.E., Diesmann, M., Gewaltig, M.O., Morrison, A. (2013). NEST: the neural simulation tool. In Jaeger, D, & Jung, R (Eds.) *Encyclopedia of Computational Neuroscience*. Berlin: Springer, DOI 10.1007/SpringerReference_348323, (to appear in print).

[CR52] Richardson MJE (2007). Firing-rate response of linear and nonlinear integrate-and-fire neurons to modulated current-based and conductance-based synaptic drive. Physical Review E.

[CR53] Richardson MJE, Swarbrick R (2010). Firing-rate response of a neuron receiving excitatory and inhibitory synaptic shot noise. Physical Review Letters.

[CR54] Rotter S, Diesmann M (1999). Exact digital simulation of time-invariant linear systems with applications to neuronal modeling. Biological Cybernetics.

[CR55] Roxin A (2011). The role of degree distribution in shaping the dynamics in networks of sparsely connected spiking neurons. Frontiers in Computational Neuroscience.

[CR56] Sakata S, Harris KD (2012). Laminar-dependent effects of cortical state on auditory cortical spontaneous activity. Frontiers in Neural Circuits.

[CR57] Shimazaki H, Shinomoto S (2010). Kernel bandwidth optimization in spike rate estimation. Journal of Computational Neuroscience.

[CR58] Song S, Sjöström P, Reigl M, Nelson S, Chklovskii D (2005). Highly nonrandom features of synaptic connectivity in local cortical circuits. PLoS Biology.

[CR59] Stein RB (1965). A theoretical analysis of neuronal variability. Biophysical Journal.

[CR60] Tuckwell HC (1988). Introduction to theoretical neurobiology, Vol. 1.

[CR61] Weber AI, Pillow JW (2017). Capturing the dynamical repertoire of single neurons with generalized linear models. Neural Computation.

[CR62] Wilson HR, Cowan JD (1972). Excitatory and inhibitory interactions in localized populations of model neurons. Biophysical Journal.

[CR63] Wolfram S (1999). The mathematica book.

[CR64] Yamauchi S, Kim H, Shinomoto S (2011). Elemental spiking neuron model for reproducing diverse firing patterns and predicting precise firing times. Frontiers in Computational Neuroscience.

